# SATB2 Mediates H3K9 Delactylation by Recruiting HDAC3 to Repress LCN2 and Inhibit Lung Tumor Growth and Metastasis

**DOI:** 10.1002/advs.202522996

**Published:** 2026-02-25

**Authors:** Ting Wen, Lihua Yang, Shuang Cai, Yunxia Liu, Jianan Fan, Peng Wu, Beibei Gao, Xiaoge Xie, Hong Sun, Sida Qin, Qiao Yi Chen

**Affiliations:** ^1^ Department of Cell Biology and Genetics School of Basic Medical Sciences Xi'an Jiaotong University Xi'an Shaanxi China; ^2^ Centre for Chromosome Biology School of Natural Sciences University of Galway Galway Ireland; ^3^ Division of Environmental Medicine Department of Medicine New York University Grossman School of Medicine New York New York USA; ^4^ Department of Thoracic Surgery The First Affiliated Hospital of Xi'an Jiaotong University Xi'an Shaanxi China

**Keywords:** H3K9 delactylation, HDAC3, LCN2, lung cancer, SATB2

## Abstract

Lung cancer remains a leading cause for global cancer‐related mortality, with therapeutic resistance and metastasis posing major clinical challenges. The special AT‐rich sequence‐binding protein 2 (SATB2) is a well‐established tumor suppressor in NSCLC, but its downstream epigenetic and metabolic regulatory mechanisms remain largely unclear. Here, we demonstrate that SATB2 exerts tumor‐suppressive effects by impairing NSCLC cell proliferation, migration, invasion, and EMT. Mechanistically, SATB2 functions as a negative regulator of global histone lactylation, with a specific role in reducing histone H3 lysine 9 lactylation (H3K9la)—a previously uncharacterized histone mark in NSCLC. Through integrated multi‐omics analyses (RNA‐seq and H3K9la‐specific CUT&Tag), we identified Lipocalin‐2 (LCN2), an oncoprotein, as a critical downstream target of the SATB2‐H3K9la axis. SATB2 is able to bind LCN2 promoter and recruit histone deacetylase 3 (HDAC3) via its N‐terminal domain, catalyzing H3K9 delactylation to repress LCN2 transcription. Exogenous lactate reversed SATB2‐mediated H3K9la and LCN2 suppression, restoring oncogenic phenotypes. In vivo, SATB2 overexpression inhibited xenograft tumor growth and lung metastasis, while LCN2 overexpression rescued these suppressive effects. Our findings uncover a novel epigenetic‐metabolic crosstalk pathway in NSCLC, providing new insights into the molecular mechanisms of SATB2‐mediated tumor suppression and potential therapeutic targets for NSCLC.

## Graphical abstract

This study highlights the capacity for tumor suppressor SATB2 to recruit HDAC3 and catalyze histone H3K9 delactylation, thereby repress transcription of the oncogene LCN2. This novel epigenetic‐metabolic axis inhibits the growth and metastasis of non‐small cell lung cancer, delineating a new mechanistic pathway with potential therapeutic relevance.

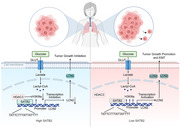

## 1 Introduction

Lung cancer is the leading cause of cancer‐related mortality worldwide, representing a major global public health challenge [1]. As the most frequently occurring cancer (14.3%), the incidence and mortality of lung cancer in regions of high human development index (HDI) is approximately three times greater than that of regions with low HDI [2]. Lung cancer is classified into small cell lung cancer (SCLC), and non‐small cell lung cancer (NSCLC), including adenocarcinoma, squamous cell carcinoma, and large cell carcinoma, which together accounts for 85% of all lung cancer cases. Despite tremendous improvement in prognosis due to technological advances in early screening and therapeutic treatments, development of therapeutic resistance and late‐stage metastasis remain major challenges. Epithelial‐mesenchymal transition (EMT) for instance, plays a fundamental role in empowering cancer cells to enhance the migratory capacity, invade surrounding tissues, and spread to distant organs. Moreover, the inactivation of key tumor suppressor genes, which normally function as gatekeepers of cell proliferation and apoptosis, is equally important as the activation of tumor promoting genes in lung tumorigenesis. Furthermore, the crosstalk between epigenetic and metabolic reprogramming adds further complexity for driving lung tumor progression.

The special AT‐rich sequence‐binding protein 2 (SATB2) gene encodes for a nuclear matrix attachment region (MAR) protein that functions as a critical transcriptional regulator via higher‐order chromatin organization [3, 4]. As a member of the SATB family, SATB2 shares similar homology and function with SATB1, both of which facilitate transcription regulation via chromatin remodeling [5]. While initially identified as being involved in craniofacial morphogenesis, increasing evidence has pointed toward the role of SATB2 in tumor development, including lung, colorectal, bone, and oral squamous cell carcinoma [3, 6]. In lung cancer, SATB2 is a well‐established tumor suppressor, especially in inhibiting EMT [7, 8, 9, 10, 11, 12, 13]. While currently available literature has primarily elucidated the upstream regulation of SATB2‐identifying repressors such as miR‐31‐5p, miR‐34c‐5p, miR‐875‐5p, miR‐660, and long non‐coding RNA HCG11, which have been evidenced to promote lung cancer progression via downregulating SATB2 expression–the downstream effector pathways through which SATB2 exerts its tumor‐suppressive functions remain largely elusive [10, 11, 12, 13, 14]. Thus, despite compelling evidence demonstrating the tumor suppressive role of SATB2 in lung cancer, the precise downstream molecular mechanisms warrant further investigation.

The eukaryotic chromatin is composed of basic repeating nucleosome subunits, consisting of a histone octamer (H2A, H2B, H3, and H4), wrapped around 147 bp DNA in a bead on string fashion. In‐depth effort to map the human epigenome has unveiled numerous epigenetic modifications on the terminal tail of nucleosomes, including acetylation, methylation, SUMOylation, ubiquitination, and phosphorylation [15]. Lactylation is a newly discovered post‐translational modification that consists of the addition of a lactyl (La) group on lysine ε‐amino acid residues to epigenetically regulate gene expression, linking cellular metabolism to epigenetic regulation [16]. Notably, the level of histone lactylation is dependent on the availability of lactate, an end product of glycolysis. The long‐standing perception of lactate as a simple waste product was redefined by the seminal study led by DeBerardinis et al., which demonstrated that lactate serves as a vital nutrient for cancer cells [17, 18]. Moreover, it is now clear that the high intracellular pool of lactate found in the tumor milieu is a source of precursors for epigenetic signaling, thereby facilitating tumor progression. In the context of cancer, the involvement of histone lactylation has been reported in non‐small cell lung cancer, ocular melanoma, pancreatic adenocarcinoma, gastric cancer, hepatocellular carcinoma, and melanoma [19, 20, 21, 22, 23, 24]. For lung cancer in particular, histone H3K18, H3K14, H4K8, H4K16, H4K12 lactylation has been reported [25, 26, 27, 28, 29]. Similar to histone acetylation, lactylation is modified by “writers” (p300, KAT8, AARS2) and “erasers” (class I‐III histone deacetylases, HDAC1‐3) [17, 30, 31]. Although current studies have primarily focused on the direct effect of dysregulated glycolysis in promoting histone lactylation, specific mechanisms governing histone delactylation remain largely unexplored in lung cancer. Interestingly, SATB2 has been evidenced to interact with HDACs in the brain, small intestine, and skeletal muscle [32, 33, 34, 35, 36]. However, the functional relevance of SATB2‐HDAC interaction and their potential role in influencing the epigenetic‐metabolic crosstalk in lung cancer warrant further investigation.

In this study, we discovered the novel role of SATB2 in facilitating global and histone H3K9‐specific delactylation through HDAC3 recruitment, subsequently inhibiting the expression of oncoprotein Lipocalin‐2 (LCN2). Notably, we identified H3K9la as novel histone mark whose regulation in lung cancer has not been previously characterized. In addition, we established in vivo models demonstrating that the tumor‐suppressive effects of SATB2 on both xenograft tumor growth and metastasis are effectively rescued by LCN2 overexpression. Our findings collectively provide a new perspective on the epigenetic‐metabolic crosstalk in lung tumorigenesis.

## 2 Results

### 2.1 SATB2 Exerts Tumor‐Suppressive Functions in Lung Cancer Cells by Impairing Proliferation, Migration, and Invasion

**FIGURE 1:**
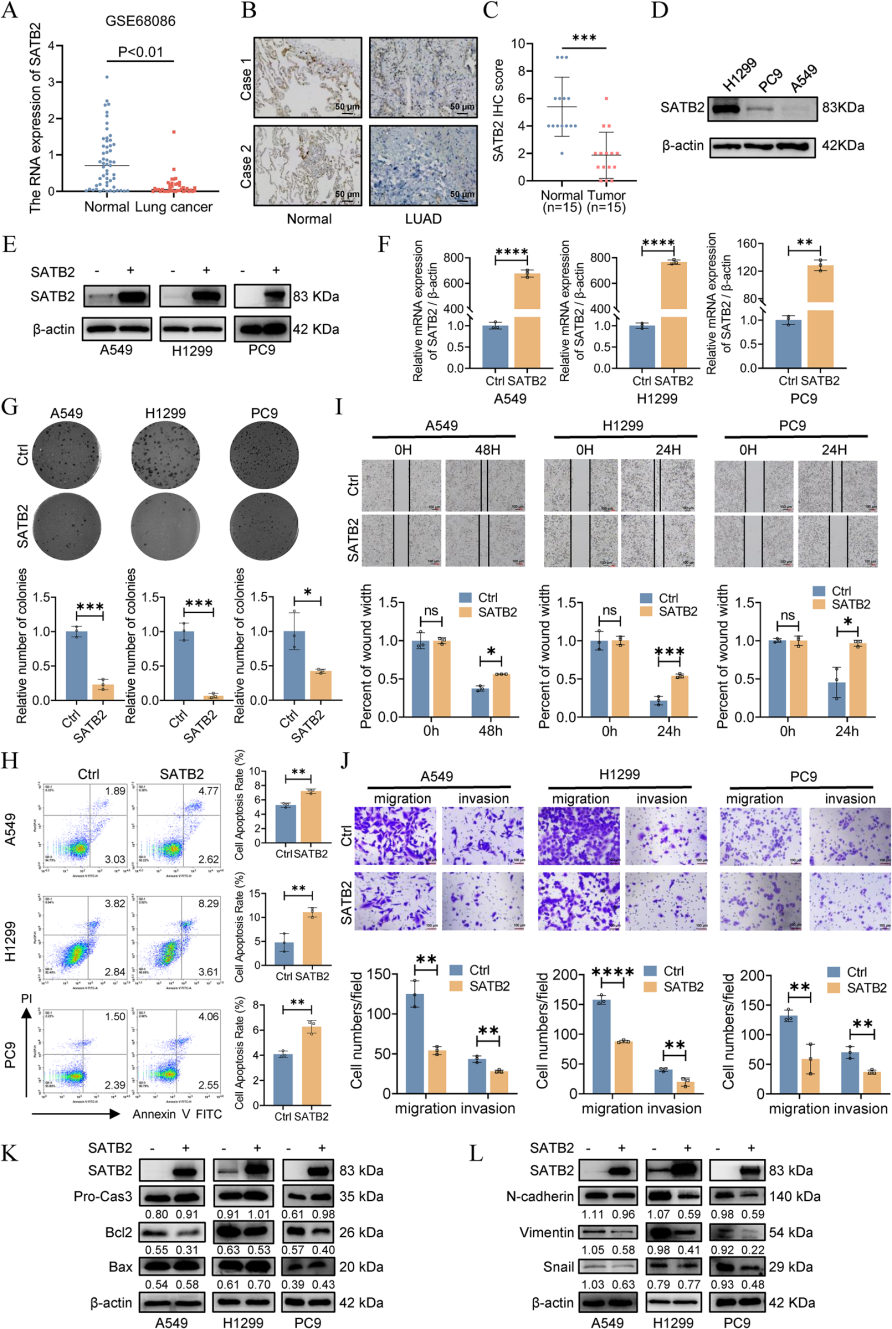
SATB2 inhibits proliferation and migration of lung cancer cells. A. Comparison of SATB2 mRNA expression in normal and lung cancer patients (GSE68086. *n* = 94, Welch's *t*‐test). B. Representative immunohistochemistry images showing SATB2 expression in normal lung tissues and LUAD tissues from patients. C. The quantification of 15 matched IHC staining of LUAD tissues (tumor) and adjacent normal tissues (normal) (paired *t*‐test). D. Protein expression levels of LUAD cells. E,F. A549, H1299 and PC9 cells were transiently transfected with SATB2 overexpression vector (SATB2) or control vector (Ctrl). SATB2 protein and mRNA levels were measured using western blotting assays (E) and qRT‐PCR (F). G. SATB2 suppressed the cell colony formation ability. H. The cell apoptosis rate was measured using flow cytometry. I,J. The migration ability was evaluated by wound healing and transwell assays. K,L. The expression of apoptosis molecular and EMT markers were measured by western blotting assays. *P < 0.05; **P < 0.01; ***P < 0.001; ns, no significance.

Substantial evidence from numerous studies have established SATB2 as a tumor suppressor in lung cancer [3, 7, 8, 9, 10, 11, 12, 13]. To interrogate its clinical relevance, we assessed SATB2 expression by analyzing GEO database (GSE68086) and patient‐derived samples via immunohistochemistry (IHC). As shown in Figure 1A–C, SATB2 expression is aberrantly downregulated in lung cancer patients compared with adjacent normal tissues. Interestingly, the expression level of SATB2 varies among different lung cancer cell lines. Specifically, A549 and PC9 cells showed lower SATB2 expression compared to H1299 cells (Figure 1D; Figure S1A). To elucidate the functional consequences of SATB2 in vitro, we generated SATB2 overexpression plasmid and transfected it into A549, H1299, and PC9 cells. Transfection efficiency was confirmed by western blotting and qRT‐PCR, which verified successful and significant SATB2 upregulation at both protein and transcript levels (Figure 1E,F). Subsequent functional assessment using colony formation assay demonstrated that SATB2 overexpression markedly impaired clonogenic survival, resulting in a significant reduction in the number of colonies formed compared to control cells (Figure 1G). Next, we employed flow cytometry experiments to investigate the function of SATB2 overexpression on cell apoptosis. As shown in Figure 1h, SATB2 overexpressing cells demonstrated a substantially higher apoptotic rate compared to their control counterparts. For further validation, we performed wound healing assays to assess the impact of SATB2 on cellular motility. As expected, results indicated that SATB2 overexpression significantly inhibited cell migration relative to the control group (Figure 1I; Figure S1B). Corroborating these findings, transwell migration and invasion assays demonstrated that SATB2 overexpression potently suppressed the migratory and invasive capacities of A549, H1299, and PC9 cells (Figure 1J). Given the pro‐apoptotic role of SATB2, we profiled the expression of key apoptotic markers. Specifically, western blotting analysis indicated that SATB2 upregulation enhanced the expression of pro‐apoptotic factors including Bax and caspase‐3 (Cas3), while concurrently suppressing the anti‐apoptotic protein Bcl‐2 (Figure 1K; Figure S1C). These findings establish the role of SATB2 in inhibiting the migratory and invasive phenotypes of lung cancer cells. For further discovery, we examined the expression of EMT markers. Western blotting analyses revealed that the ectopic expression of SATB2 attenuated the protein levels of key EMT markers, including N‐cadherin, Vimentin, and Snail (Figure 1L), suggesting a potential mechanism for its suppressive effects.

**FIGURE 2:**
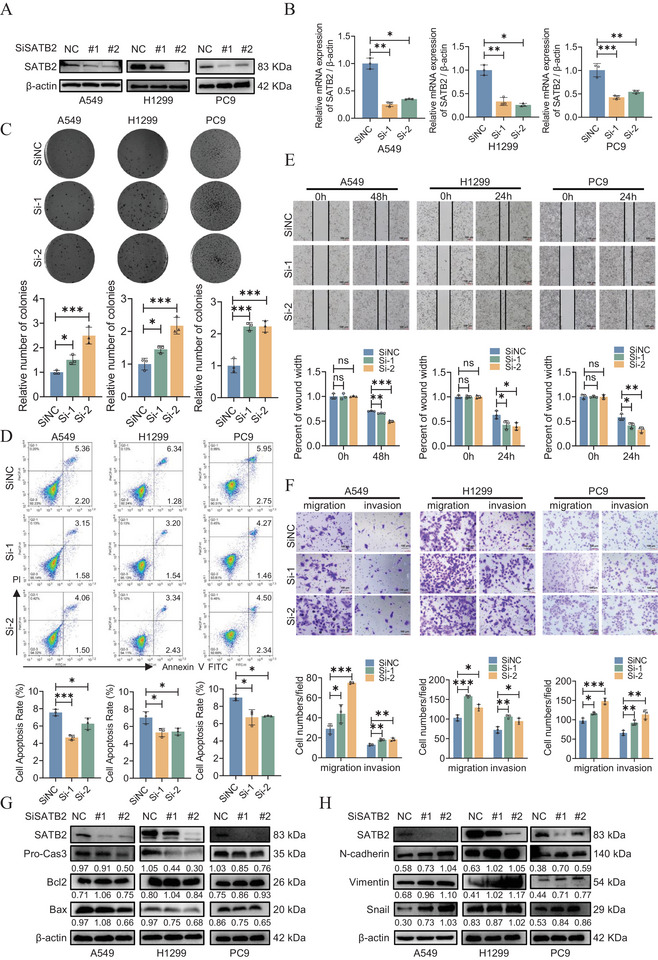
SATB2 knockdown promotes the migration and invasion of lung cancer cells. A549, H1299 and PC9 cells were transiently transfected with SATB2 siRNA (SiSATB2) or negative control siRNA (SiNC). SATB2 protein and mRNA levels were measured using western blotting assays (A) and qRT‐PCR (B). C. Inhibition of Satb2 promotes cell colony formation. D. The cell apoptosis rate was measured using flow cytometry. E,F. The migration ability was evaluated by wound healing and transwell assays. G,H. The expression of apoptosis molecular and EMT markers were measured by western blotting assays. *P < 0.05; **P < 0.01; ***P < 0.001; ns, no significance.

To corroborate the above findings, we generated SATB2 knockdown cells and confirmed the transfection efficiencies by western blotting and qRT‐PCR (Figure 2A,B). Functional assays revealed that SATB2 depletion significantly enhanced oncogenic phenotypes. Specifically, colony formation capacity was increased in knockdown cells (Figure 2C), and flow cytometry analysis indicated a pronounced reduction in the rate of apoptosis (Figure 2D). Furthermore, wound healing assays demonstrated that SATB2 knockdown potentiated cellular migration (Figure 2E; Figure S1D). This pro‐migratory phenotype was reinforced by transwell assays, which showed a significantly greater number of cells passing through the chamber in the knockdown group compared to the control group (Figure 2F). In addition, SATB2 knockdown cells showed reduced protein expressions of Bax, Cas3, but promoted Bcl‐2 levels (Figure 2G; Figure S1E). In contrast to SATB2 overexpressing cell lines, SATB2 knockdown cells demonstrated enhanced expression of N‐cadherin, vimentin, and snail (Figure 2H). Collectively, the above experiments proved that SATB2 demonstrates tumor suppressive abilities and that overexpression of SATB2 potentiates the inhibition of lung cancer cell proliferation, migration, and invasion. However, the underlying molecular mechanism, especially its downstream pathways, warrant further investigation

### 2.2 SATB2 Acts as a Negative Regulator of Global and H3K9la Lactylation in Lung Cancer

**FIGURE 3:**
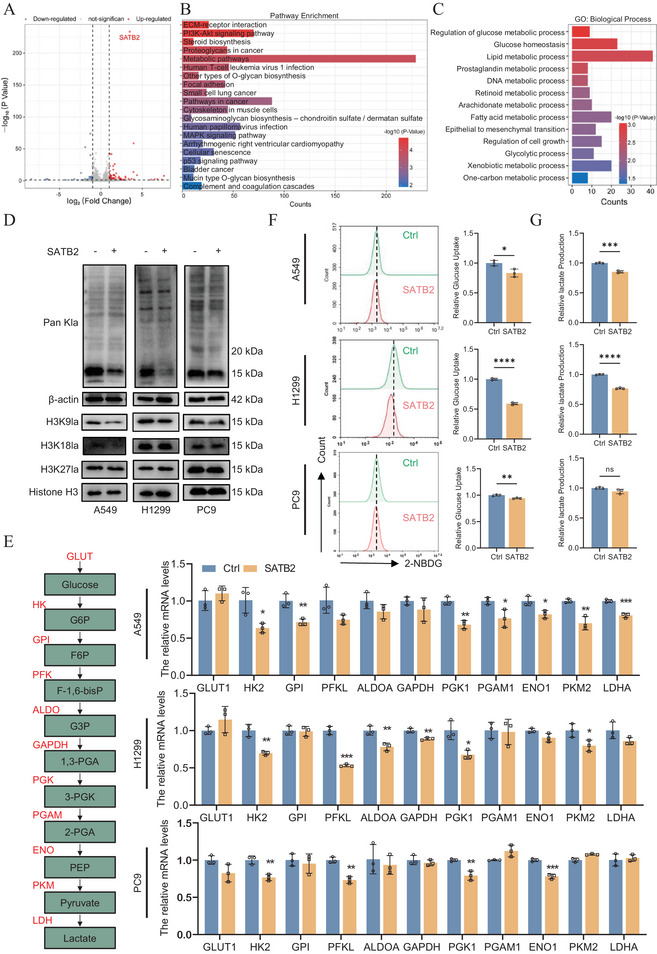
SATB2 mediates global and H3K9 delactylation. A. The volcano plot displayed differentially expressed genes in transcriptome sequencing. B. The pathway enrichment of differentially expressed genes based on RNA‐seq data. C. GO enrichment analysis of metabolism related pathways based on RNA‐seq data. Volcano plot and bar blot were plotted using Hiplot Pro (https://hiplot.com.cn/), a comprehensive web service for biomedical data analysis and visualization. D. The protein level of Pan Kla, H3K9la, H3K18la and H3K27la were measured by western blotting assays. E. qRT‐PCR analysis of glycolytic gene expression in SATB2 overexpression cells. F,G. SATB2 inhibits glucose uptake and lactate production. *P < 0.05; **P < 0.01; ***P < 0.001; ns, no significance.

Previous studies have reported the ability of SATB2 to recruit chromatin remodeling complexes and act as a transcription factor [32, 33, 34, 35, 36, 37, 38]. To gain further insight into the molecular pathways governed by SATB2, we first performed RNA‐sequencing (RNA‐Seq) using control and SATB2 overexpressing A549 cells. Our results indicated that significantly differentially expressed genes (DEGs) in SATB2 overexpressing cells were highly enriched in oncogenic and metabolic pathways (Figure 3A–C; Figure S2A,B). Interestingly, through KEGG and GO analyses, we observed pronounced enrichment in genes and pathways related to glucose and glycolysis metabolism (Figure S2C–E). Moreover, specific enriched pathways such as PI3K‐Akt and MAPK signaling pathways have been reported to be closely involved with glycolysis and protein lactylation in tumorigenesis [40, 41, 42, 43]. Moreover, a study led by Yang et al. showed that lactate dehydrogenase A (LDHA) was up‐regulated in SATB2^−/−^ mice, implying a potential role for SATB2 in modulating lactate metabolism [39]. Given these findings, we postulate that the tumor suppressive function of SATB2 in lung cancer may be mediated, at least in part, through the regulation of glycolytic processes. To validate this speculation, we first examined the effect of SATB2 expression on global protein lactylation. As shown in Figure 3D, western blotting results indicated that SATB2 overexpression significantly downregulated histone lactylation in NSCLC cells, whereas non‐histone protein lactylation was largely unaltered. This specificity prompted a focused investigation into histone lactylation. A panel of lactylation marks with known relevance in cancer was subsequently analyzed using western blotting. We focused on H3K18la and H3K27la, as these are commonly characterized lactylation marks in cancer, including in NSCLC for H3K18la. We also included H3K9la due to its emerging role in oncogenesis, despite being uncharacterized in lung cancer. As our results indicated, while overexpression of SATB2 had no significant effect on H3K18la nor H3K27la, H3K9la was markedly reduced. The potential pathological significance of H3K9la was further underscored by its elevated baseline expression across a panel of cancer cell lines (Figure S3A). Noted, future work should aim to examine a broader spectrum of lactylation marks to fully delineate the site‐specific regulatory scope of SATB2.

Given the documented crosstalk between histone lactylation and acetylation, we sought to determine the specificity of SATB2's effect. Western blotting analysis for Pan‐KAc revealed that global histone acetylation levels were unaltered by SATB2 expression (Figure S3B). These data collectively demonstrate that SATB2 specifically suppresses global histone lactylation, with a pronounced effect on H3K9 delactylation in NSCLC cells. We next sought to elucidate the mechanism underlying H3K9 delactylation. Our RNA‐seq data suggested that DEGs in SATB2 overexpressing cells are closely related with glycolysis. Thus, we began by evaluating the expression levels of key glycolytic genes in SATB2 overexpressing cells, including GLUT1, HK2, GPI, PFKL, ALDOA, PGK1, PGAM1, ENO1, PKM2, LDHA, and GAPDH. Intriguingly, our results indicated that in A549 cells, SATB2 was able to inhibit the expression of HK2, GPI, PGK1, PGAM1, ENO1, PKM2, and LDHA. However, a cross‐comparison of all three cell lines only identified HK2 and PGK1 as consistently repressed targets by SATB2 overexpression (Figure 3E). Next, we verified whether SATB2 has an effect on glucose uptake and lactate production. As shown in Figure 3F,G, SATB2 overexpression negatively regulated glycolysis in A549 and H1299 cells, but not in PC9 cells. This critical observation suggested that SATB2‐mediated H3K9 delactylation may occur through a mechanism independent of, or in addition to, the inhibition of lactate synthesis. Collectively, our findings establish SATB2 as a negative regulator of global Kla and H3K9la in NSCLC cells. Importantly, we provide evidence that this effect may not simply be a consequence of direct inhibition of glycolysis, pointing to a more specific pathway for histone delactylation.

### 2.3 SATB2 Represses LCN2 Transcription by Diminishing H3K9la Occupancy at Its Promoter

**FIGURE 4:**
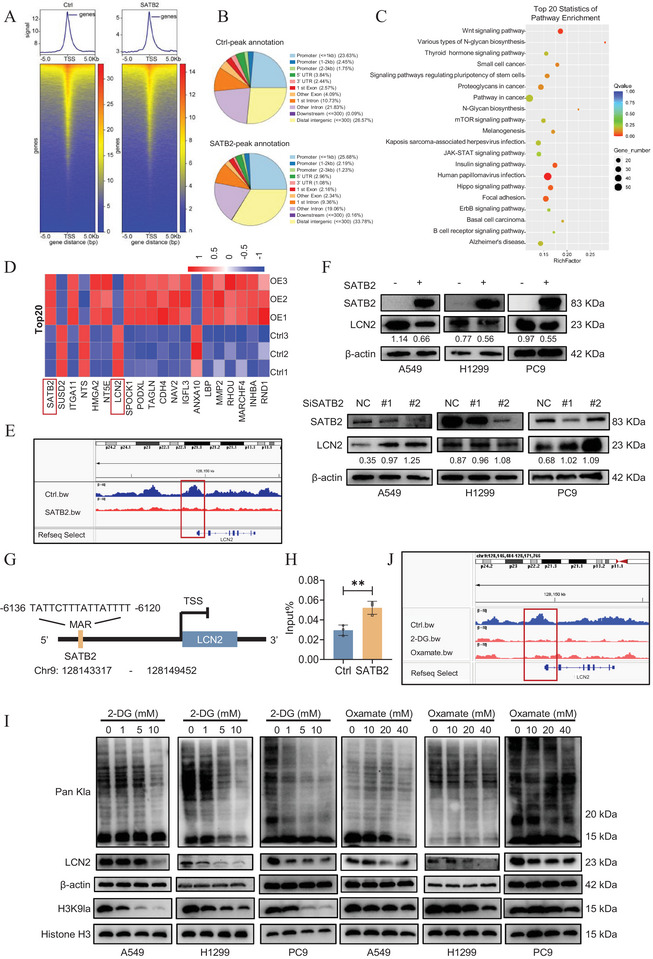
SATB2 transcriptionally represses LCN2 by inhibiting H3K9la deposition. A,B. CUT&Tag was performed with H3K9la antibodies in A549 cells. The heatmap showed the distribution of H3K9la peaks in the transcriptional start site (TSS) (A). The distribution of H3K9la on the genome (B). C. KEGG database analysis for H3K9la‐related genes. D. Transcriptome sequencing was performed in SATB2‐overexpressing A549 cells. The heatmap showed top 20 differentially expressed genes. E. IGV views of H3K9la CUT&tag sequence data on LCN2. F. LCN2 protein levels were detected using western blotting assays. G. Schematic representation of MAR (Matrix Attachment Region) within LCN2 locus. H. ChIP‐qPCR of A549 cells stably overexpressing SATB2 using SATB2 antibody. I. Cells were treated with 2‐DG or oxamate (glycolysis inhibitors) at different concentrations for 24 h. Cells were collected for measuring the level of Pan Kla, H3K9la and LCN2. J. IGV views of H3K9la CUT&tag sequence data on LCN2 with glycolysis inhibitors treated. *P < 0.05; **P < 0.01; ***P < 0.001; ns, no significance.

The above experiments confirmed the tumor suppressive function of SATB2 and its role in mediating H3K9 delactylation in NSCLC cells. Next, we sought to delineate the regulatory mechanism of H3K9 delactylation and identify critical downstream effectors of SATB2. To this end, we performed H3K9la‐specific CUT&Tag assays in conjunction with RNA‐seq in SATB2 overexpressing A549 cells. Genomic distribution analysis revealed a significant enrichment of H3K9la peaks within promoter regions of target genes (Figure 4A,B). Interestingly, the enrichment of H3K9la in the promoter region was significantly reduced in the SATB2 overexpressing group compared to the control group. Subsequent KEGG database analysis of genes associated with H3K9la peaks indicated enrichment in tumorigenesis‐related signaling pathways, including general pathways in cancer as well as pathways involving Hippo, JAK‐STAT, mTOR, and Wnt signaling (Figure 4C). Through an integrative multi‐omics approach, we performed a systematic screening using CUT&Tag, RNA‐Seq, and qRT‐PCR validation, supplemented by PubMed literature search, and identified Lipocalin‐2(LCN2) as a principal downstream target co‐regulated by both SATB2 and H3K9la lactylation (Figure 4D,E; Figure S4A–E). CUT&Tag analysis revealed reduced H3K9la occupancy at the LCN2 promoter in SATB2 overexpressing cells (Figure 4E), a finding consistent with the inverse correlation found between SATB2 and LCN2 expression in RNA‐Seq data. This repressive relationship was further confirmed at the protein and RNA levels, where overexpression of SATB2 suppressed LCN2 expression across all three cell lines (Figure 4F; Figure S5A). Reciprocally, SATB2 knockdown elevated LCN2 expression, confirming SATB2 as a transcriptional repressor of LCN2.

Based on the findings above, we hypothesized that SATB2 represses LCN2 expression by diminishing H3K9la occupancy at its promoter. To test this hypothesis, we first evaluated potential binding between SATB2 and LCN2. Marscan was used to search for MAR recognition signatures‐specific 8 bp (AATAAYAA) and 16 bp (AWWRTAANNWWGNNNC) sequences within a 200 bp window [44]‐we identified a putative SATB2 binding site (6136‐TATTCTTTATTATTTT‐6121) upstream of the LCN2 promoter (Figure 4G). Binding between SATB2 and LCN2 was subsequently validated through chromatin immunoprecipitation quantitative PCR (ChIP‐qPCR) (Figure 4H). Next, based on the finding that SATB2 attenuates H3K9la occupancy at the LCN2 promoter, we aimed to determine whether the reduction in H3K9la lactylation is sufficient to suppress LCN2 expression. To directly inhibit histone lactylation, we treated A549, H1299, and PC9 cells with glycolysis inhibitors 2‐deoxyglucose (2‐DG) and oxamate (Figure S5B). As our data indicated, treatment with 2‐DG and oxamate significantly reduced H3K9la lactylation as well as LCN2 expression levels in a dose‐dependent manner (Figure 4I; Figure S5C). Additionally, CUT&Tag analyses revealed that this pharmacological inhibition significantly reduced H3K9la occupancy at the LCN2 promoter (Figure 4J; Figure S5D,E). In addition, luciferase reporter assays were performed to examine whether lactate supplementation promoted LCN2 promoter activity. As shown in Figure S5F,G, while SATB2 overexpression inhibited luciferase activity, NALA treatment significantly increased LCN2 promoter activity. Based on these correlative findings, we propose that SATB2‐mediated repression of LCN2 is linked to the specific reduction of H3K9 lactylation at its promoter. We note that the precise causal relationship requires further validation through targeted epigenetic editing.

### 2.4 LCN2 Overexpression Rescues the Tumor‐Suppressive Phenotypes of SATB2 in Lung Cancer

**FIGURE 5:**
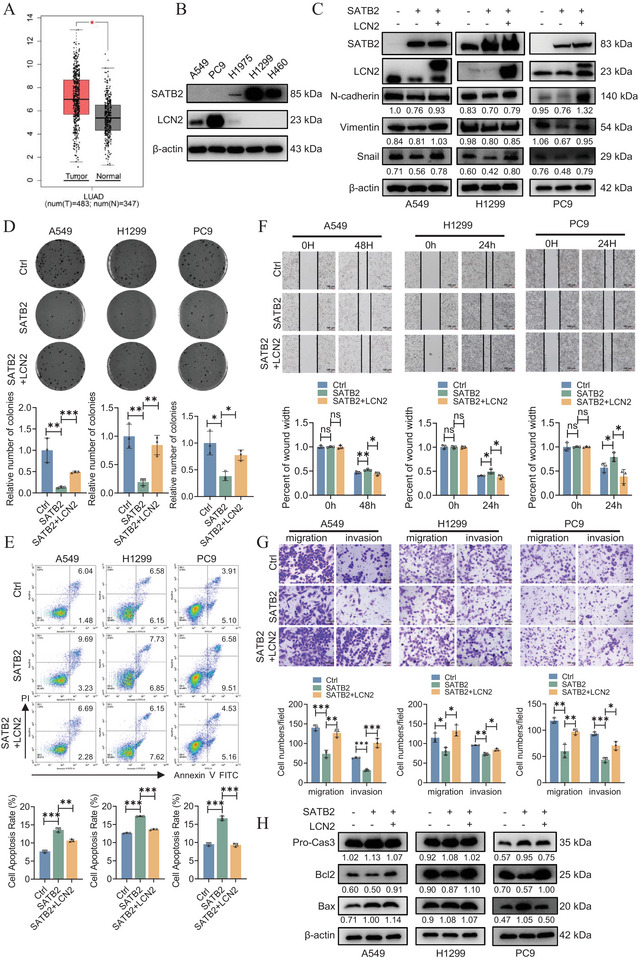
Ectopic expression of LCN2 rescues the tumor‐suppressive phenotypes induced by SATB2. A. LCN2 mRNA was upregulated in LUAD in the TCGA database (using the online database GEPIA). B. The protein expression of SATB2 and LCN2 in normal cells and NSCLC cells. C. A549, H1299 and PC9 cells were transiently transfected with SATB2 overexpression vector (SATB2) and LCN2 overexpression vector (LCN2). SATB2 and LCN2 protein levels were measured using western blotting assays. D. Cell proliferation and colony formation ability were measured by colony formation assay. E. The cell apoptosis rate was measured using flow cytometry. F,G. The migration ability was evaluated by wound healing and transwell assays. H. The expression of apoptosis molecular (H) and EMT (C) markers were measured by western blotting assays. *P < 0.05; **P < 0.01; ***P < 0.001; ns, no significance.

Emerging evidence points toward the role of LCN2 in lung tumor progression [45, 46, 47, 48, 49]. To substantiate the function of LCN2 in NSCLC, we performed TCGA analysis, which indicated a significant overexpression of LCN2 in lung cancer tissues compared to normal controls. (Figure 5A; Figure S6A). As the putative downstream target of SATB2, the expression levels of LCN2 were also examined in lung cancer cell lines. As expected, the expression levels of SATB2 and LCN2 in lung cancer cells are inversely correlated (Figure 5B). Next, rescue experiments were carried out to verify the role of SATB2 and LCN2 in lung cancer cell progression and invasion. Notably, LCN2 expression was found inversely correlated with SATB2 in lung cancer cells. Thus, we overexpressed LCN2 in cells overexpressing SATB2, and determined the expression of EMT‐related markers using western blotting. Our results indicated that while SATB2 overexpression reduced EMT‐related markers (N‐cadherin, Vimentin, and Snail), LCN2 overexpression led to partially enhanced expression levels (Figure 5C; Figure S6B), indicating opposing regulatory roles. Furthermore, while SATB2 overexpression potently inhibited clonogenic growth, co‐expression of LCN2 significantly restored colony formation capacity (Figure 5D). Similarly, the pro‐apoptotic effect of SATB2 was markedly attenuated by LCN2 overexpression (Figure 5E). In wound healing assays, the migratory suppression imposed by SATB2 was counteracted by concomitant LCN2 overexpression, indicating that LCN2 is epistatic to SATB2 in this process (Figure 5F; Figure S6C). Furthermore, the pro‐migratory and invasive phenotype was further corroborated by transwell assays (Figure 5G). Lastly, western blotting analyses of apoptosis‐related markers provided additional mechanistic support for these findings (Figure 5H; Figure S6D). Altogether, these results confirmed LCN2 as a tumor‐promoting factor that is both repressed by SATB2 and functionally capable of rescuing the tumor‐suppressive effects of SATB2 in lung cancer cells.

### 2.5 Exogenous Lactate Reverses SATB2 and LDHA‐Mediated Suppression of LCN2 and Tumor Phenotypes

**FIGURE 6:**
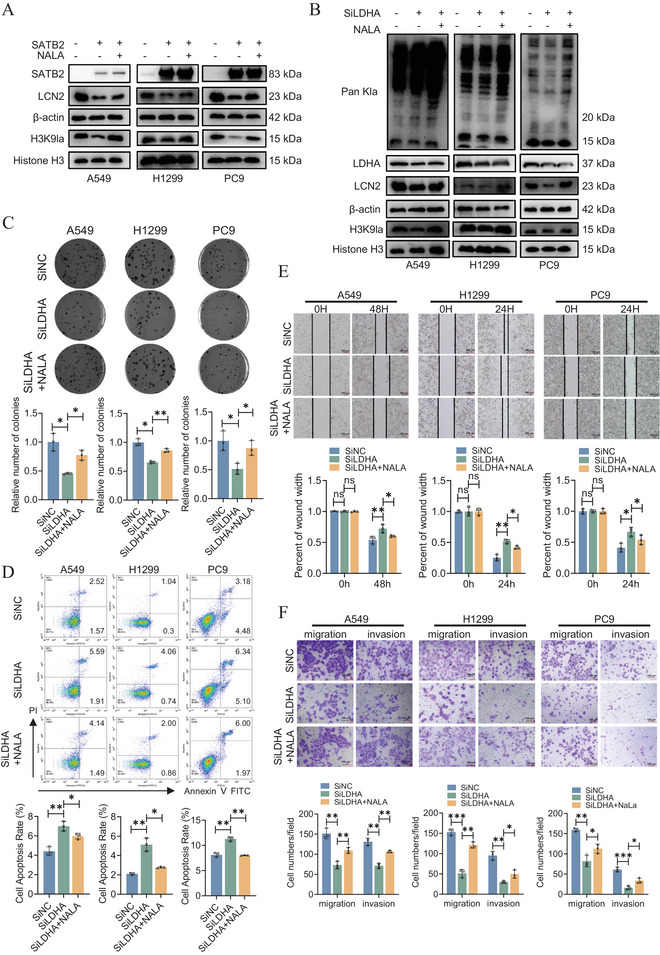
Sodium lactate supplementation rescues LCN2 expression. A,B. The expression of LCN2 and H3K9la were measured by western blotting assays. C. Cell proliferation and colony formation ability were measured by colony formation assay. D. The cell apoptosis rate was measured using flow cytometry. E,F. The migration ability was measured by wound healing and transwell assays. *P < 0.05; **P < 0.01; ***P < 0.001; ns, no significance.

In previous experiments, we demonstrated that SATB2 binding and glycolytic inhibition suppress LCN2 expression by reducing H3K9la occupancy at its promoter. To reinforce our findings, supplementation with sodium lactate (NaLa) was used to ascertain the potential rescue effects of lactate treatment on LCN2 expression and H3K9la lactylation. Specifically, we treated SATB2 overexpressing cells with NaLa and measured LCN2 and H3K9la via western blotting. As shown in Figure 6A, NaLa treatment effectively restored both LCN2 protein and H3K9la levels in SATB2 overexpressing cells. To further corroborate the central role of lactate in this axis, we performed LDHA knockdown in SATB2 overexpressing cells. As expected, while si‐LDHA cells demonstrated reduced LCN2 and H3K9la levels, NaLa treatment was able to reverse and restore their expression levels (Figure 6B; Figure S7A). Noted, the effect of LDHA knockdown on global protein lactylation was less pronounced compared to global histone lactylation. This suggests that the lactylation state of histones may be particularly sensitive to perturbations in the intracellular lactate pool. To achieve discernible effect on global non‐histone lactylation, a higher degree of LDHA knockdown and lactate pool depletion may be required to significantly impact a broader array of protein substrates. In addition, while 2‐DG and oxamate treatment inhibited LCN2 mRNA levels, the addition of NaLa reversed this effect (Figure S7B). Next, we assessed the functional consequences of this metabolic rescue. In particular, NaLa treatment significantly restored the clonogenic capacity impaired by LDHA knockdown (Figure 6C). Flow cytometry analysis revealed that the increase in apoptosis following LDHA knockdown was markedly attenuated by NaLa co‐treatment (Figure 6D). In addition, wound healing assays indicated that NaLa treatment restored the migratory deficiency in si‐LDHA cells (Figure 6E; Figure S7C). Similar results were reinforced in invasion and migration assays (Figure 6F). These data validate that exogenous lactate is sufficient to reinstate H3K9la and LCN2 levels, as well as the oncogenic phenotypes.

### 2.6 SATB2 Recruits HDAC3 to Catalyze H3K9 Delactylation and Repress LCN2 Expression

**FIGURE 7:**
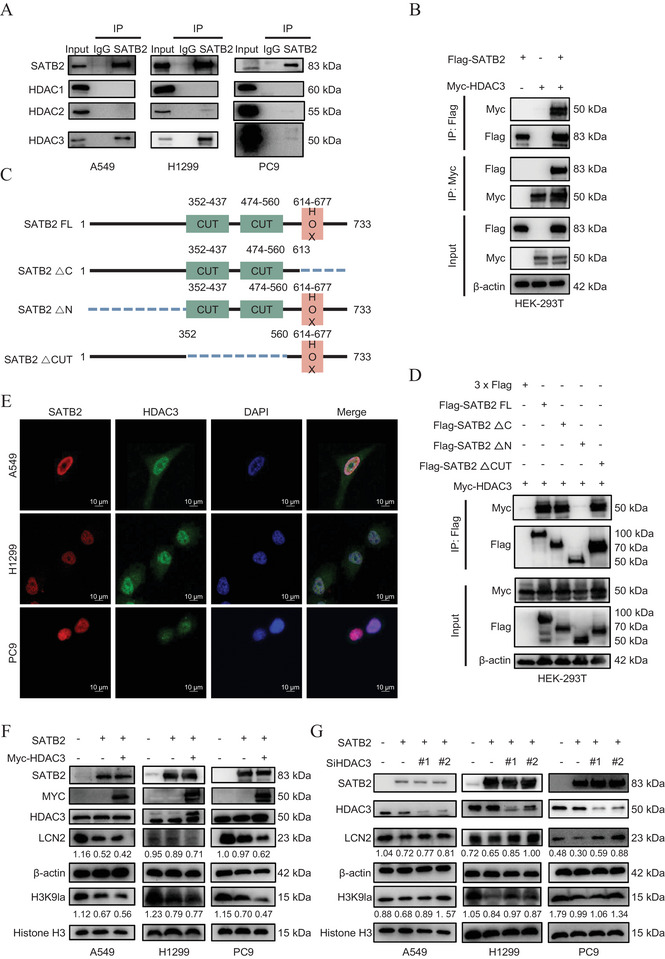
SATB2 recruits HDAC3 to mediate H3K9 delactylation and repress LCN2 expression. A. Endogenous immunoprecipitation (IP) with anti‐SATB2 in the A549, H1299 and PC9 cells followed by western blotting. IgG was used as a negative control. B. Exogenous IP of SATB2 and HDAC3 in 293T cells. C. Wild‐type SATB2 and SATB2 deletion mutants schematics. D. 293T cells transfected with Myc‐tagged HDAC3 and Flag‐tagged wild‐type SATB2 or SATB2 deletion mutants then harvested for immunoprecipitation. E. Immunofluorescence staining of SATB2 (red) and HDAC3 (green) in NSCLC cells; Cell nuclei were stained with DAPI (blue). F,G. The expression of LCN2 and H3K9la after SATB2 and HDAC3 overexpression or SiHDAC3 in cells were measured by western blotting assays. *P < 0.05; **P < 0.01; ***P < 0.001; ns, no significance.

Building on our prior evidence implicating the role of SATB2 in promoting H3K9 delactylation and reducing its occupancy at the LCN2 promoter, we aimed to understand the precise mechanistic basis for this regulation. Given that SATB2 binding alone does not directly suppress LCN2 transcription, but rather inhibit H3K9la occupancy at its promoter, we reasoned that it likely functions as a scaffold, recruiting co‐repressor complexes to mediate histone delactylation. Prior literature has documented the capacity for SATB2 to interact with histone deacetylases (HDACs) [32, 33, 34, 35, 36]. More importantly, recent studies have identified specific HDACs as enzymatic erasers of histone lactylation [30, 50, 51, 52, 53, 54, 55, 56]. Thus, we hypothesized that SATB2 is capable of recruiting HDAC complexes to the LCN2 promoter to catalyze H3K9 delactylation, thereby facilitating transcriptional repression. To confirm this speculation, we first performed co‐immunoprecipitation (Co‐IP) assays and found that SATB2 can bind with HDAC2 and HDAC3, both known as regulators of histone lactylation [30, 51, 52, 56], in A549, H1299, and PC9 cells (Figure 7A). To confirm the binding relationship between SATB2 and HDAC2‐3, Myc‐tagged HDAC2‐3 and Flag‐tagged SATB2 were separately transfected in to HEK‐293 T cells. Co‐IP results showed that Flag‐SATB2 was capable of binding to Myc‐HDAC3 (Figure 7B), underlying the selectivity of this molecular partnership.

To map the specific domain responsible for the SATB2‐HDAC3 interaction, we generated a series of deletion mutants: SATB2 ∆C, SATB2 ∆N, and SATB2 ∆CUT (Figure 7C). Co‐IP results indicated that the SATB2 ∆N (1‐352) region was required for HDAC3 binding, as this interaction was abolished in the SATB2 ∆N mutant but retained in the SATB2 ∆ C (613‐733) and SATB2 ∆CUT (352‐560) constructs (Figure 7D). To examine whether histone H3 mediates the interaction between SATB2 and HDAC3, we performed endogenous Co‐IP and demonstrated that histone H3 was able to co‐precipitate with HDAC3, but not SATB2, suggesting that their association is unlikely to be bridged by H3 (Figure S7D). However, these data cannot exclude the involvement of other potential bridging factors, and future in vitro binding assays with purified proteins are warranted to further clarify the binding mechanism. In addition, we carried out immune‐fluorescence assays to identify the location of SATB2 and HDAC3 co‐localization in lung cancer cells. As shown in Figure 7E, SATB2 and HDAC3 co‐localized primarily in the nucleus. To further verify the functional role of HDAC3, we modulated its expression in lung cancer cells via transfection with either Myc‐HDAC3 or si‐HDAC3. Consistent with HDAC3 functioning as a lactylation eraser, its overexpression in SATB2 overexpressing cells further suppressed LCN2 expression and H3K9la levels (Figure 7F,G). Conversely, HDAC3 knockdown cells demonstrated elevated LCN2 expression and H3K9la levels in SATB2 overexpressing cells. In addition, GEO database (GSE30219) analysis demonstrated that LCN2 is negatively correlated with both SATB2 and HDAC3, and that SATB2 and HDAC3 are positively correlated (Figure S7E). Moreover, western blotting analysis of control and patient tissue samples demonstrated positive correlation between SATB2 and HDAC3, as well as an inverse relationship between SATB2, LCN2, and H3K9la (Figure S7F). In summary, these results definitively characterize the physical interaction between SATB2 and HDAC3, mediated by the SATB2 N‐terminal domain, and establish HDAC3 as the critical enzymatic effector through which SATB2 promotes H3K9 delactyation to repress LCN2 transcription.

### 2.7 LCN2 Overexpression Antagonizes the Suppressive Effects of SATB2 on Tumor Growth and Metastasis In Vivo

**FIGURE 8:**
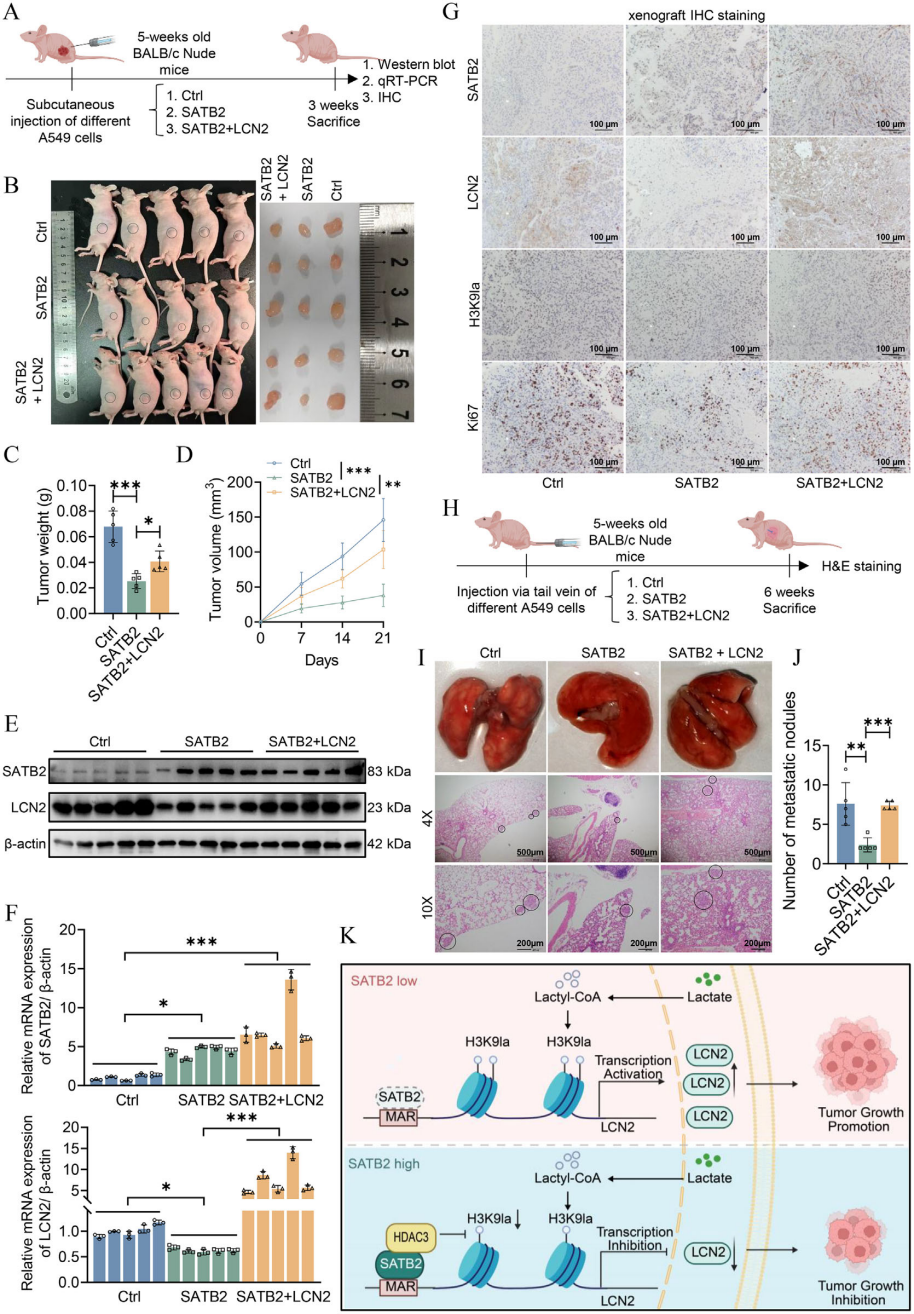
The SATB2‐LCN2 axis inhibits tumor growth and metastasis in vivo. A,B. Twenty‐one days later, all the mice were sacrificed and their tumors were dissected and photographed (*n*  =  5 per group). C‐D. The weight and volume of the tumors were recorded. E. The expression of SATB2 and LCN2 in mice tumor tissues were measured by western blotting assays. F. The mRNA levels of SATB2 and LCN2 were measured by qRT‐PCR. G. Immunohistochemistry was used to visualize and compare the protein levels of SATB2, LCN2, H3K9la and Ki67 in mice tumors. H,I. Forty‐two days later, the mice were sacrificed and their lung tissues were photographed and subjected to H&E staining (*n*  =  5 per group). G. The number of metastatic nodules in the lungs was counted. K. Schematic illustration of the current study. *P < 0.05; **P < 0.01; ***P < 0.001; ns, no significance.

To evaluate the combined impact of SATB2 and LCN2 on tumorigenesis in vivo, we established a xenograft model by subcutaneously injecting control, ov‐SATB2, and dual ov‐SATB2+ov‐LCN2 cells into nude mice (Figure 8A). Consistent with a tumor‐suppressive role, SATB2 overexpression markedly reduced both tumor volume and weight. This suppressive effect, however, was abrogated upon concurrent overexpression of LCN2 (Figure 8B–D). Western blotting and qRT‐PCR analyses of resected tumor tissues validated an inverse correlation between SATB2 and LCN2 expression in vivo (Figure 8E,F). Furthermore, IHC staining revealed elevated SATB2 concomitant with diminished LCN2 and H3K9la levels in the ov‐SATB2 group, alongside a reduction in the proliferation marker Ki67 (Figure 8G). Notably, in the ov‐SATB2+ov‐LCN2 group, H3K9la levels remained unchanged, suggesting that H3K9 lactylation acts upstream of LCN2 expression. The restoration of Ki67 in this group indicated a return of proliferative capacity. These findings imply that SATB2 overexpression suppresses both H3K9la and Ki67 levels in vivo, and that LCN2 mediated the rescue of tumor growth suppressed by SATB2 overexpression.

To assess metastatic potential, a nude mouse lung metastasis model was established via tail‐vein injection using control, ov‐SATB2, and ov‐SATB2+ov‐LCN2 cells (Figure 8H). Quantification of metastatic lung nodules revealed a significant decrease in metastatic burden in the ov‐SATB2 group compared to controls. Conversely, co‐overexpression of SATB2 and LCN2 significantly increased the number of metastatic nodules (Figure 8I,J). Collectively, our in vivo data confirm the tumor‐suppressive function of SATB2 and underscore the potent oncogenic role of LCN2 in counteracting this phenotype.

## 3 Discussion

Substantial evidence has previously confirmed that SATB2 functions as a prominent tumor suppressor in NSCLC, particularly in inhibiting EMT [7, 8, 9, 10, 11, 12]. For example, in A549 and NCI‐H1650 cells, knockdown of SATB2 has also been shown to induce cell invasion and EMT [8]. The same research group later identified that the crosstalk between p38 and AKT pathway was necessary for determining SATB2 expression [9]. In NSCLC patients, low SATB2 expression has been shown to correlate with tumor progression and poor prognosis. Specifically, the researchers reported that SATB2 inhibited EMT primarily through downregulation of G9a, a prominent histone methyltransferase [7]. In a cohort study involving 389 NSCLC patients, SATB2 was found significantly lower in lung tumor tissues compared to normal controls [12]. The authors further established that miR‐875‐5p downregulated SATB2 to promote cancer cell invasion. Further investigation led by Su et al. validated the miR‐875/SATB2 axis, and identified lncRNA HCG11 as the upstream regulator that suppressed lung cancer cell proliferation, invasion, and migration by inhibiting miR‐875 and thereby promoting SATB2 expression [11]. In addition, exosomal miR‐31‐5p was found to promote lung cancer cell migration and invasion by negatively regulating SATB2 expression. In the mouse metastatic model, the miR‐31 inhibitor group demonstrated increased SATB2 protein level, reduced lung metastatic nodules and increased survival rate [10]. Our findings are consistent with this paradigm, confirming that SATB2 overexpression robustly suppressed cell proliferation, migration, and invasion in multiple NSCLC cell lines as well as inhibiting tumor growth and metastasis in vivo. Moreover, our study builds upon existing knowledge, and shifts focus to identify the downstream molecular mechanisms through which SATB2 exerts tumor suppressive capacities.

A key finding in our study is the capacity for SATB2 to negatively regulate global histone lactylation, with a pronounced effect on H3K9 delactylation, underscoring the specificity of SATB2 for the novel lactylation mark in lung cancer. Histone H3K9 lactylation has reportedly been associated with hepatocellular carcinoma, glioblastoma, squamous cell carcinoma, and colorectal cancer [57, 58, 59, 60]. In colorectal cancer, elevated H3K9la increased chromatin accessibility and enhanced GRAMD1A expression, thereby promote cancer cell progression [60]. On the other hand, prior studies involving histone lactylation in lung cancer have primarily focused on H3K18la, H3K4, H3K14, H4K8, H4K16, and H4K12 [25, 26, 27, 28, 29]. A study led by Zhang et al. demonstrated that H3K18la potentiated the activation of POM121, a pore membrane protein, which subsequently enhanced MYC binding with CD274 and enhancement of PD‐L1 expression [25]. More recently, Zhao et al. reported that H3K18la was enriched in the promoter of YTHDF2, which serves as an oncogene in NSCLC [29]. Upstream regulators of histone lactylation have also been investigated. For instance, tumor‐derived exosomal lncRNA miR100hg was reported to mediate H3K14 lactylation and enhance the metastatic activity of non‐stem lung cancer cells [26]. Moreover, Yan et al. reported that SLC4A7 mediated lung cancer suppression by negatively regulating lactate homeostasis and protein lactylation [61]. Additionally, tumor suppressor LKB1 was found to promote lung cancer cell senescence via inhibition of lactate production and thereby diminishing H3K8 and H4K16 lactylation [27].

In this study, through an integrated multi‐omics approach, combining RNA‐Seq with H3K9la‐specific CUT&Tag, we identified LCN2 as a critical downstream gene co‐regulated by SATB2 and H3K9la. LCN2 has been evidenced to play a tumor promoting role in lung cancer, breast cancer, oral cancer, and colorectal cancer [45, 47 ^–^ 49, 62, 63, 64, 65, 66]. Mechanistically, LCN2 promotes EMT by modulating NF‐k B. Other downstream effectors of LCN2 include MMP‐9, JAK2/STAT3, and TGFB1/CXCL5 [48, 63, 64, 66]. Upstream regulators such as ZEB1 have been documented to promote LCN2 thereby influence ferroptosis and malignant development of NSCLC [49]. Complementing earlier studies, our results identified H3K9la as a previously unreported epigenetic mark upstream of LCN2. Specifically, we demonstrated that while SATB2 is able to bind LCN2 at the MAR site, the repression of LCN2 expression was mediated by H3K9 delactylation. This model was supported by several lines of evidence. First, both SATB2 overexpression, LDHA knockdown, and pharmacological inhibition of lactylation via 2‐DG and oxamate treatments led to the reduction of H3K9la occupancy at the LCN2 promoter and inhibited its expression. Second, rescue experiments using lactate supplementation reversed these effects. Third, overexpression of LCN2 effectively counteracted the tumor‐suppressive phenotypes induced by SATB2 both in vitro and in vivo. Interestingly, while SATB2 was shown to repress the expression levels of glycolytic genes including HK2 and PGK1, as well as overall lactate production, these effects were not universal for all lung cancer cell lines. Specifically, SATB2‐mediated H3K9 delactylation was observed in PC9 cells, although glycolysis was not significantly inhibited by SATB2 overexpression. These results suggested that SATB2 played a more direct role in orchestrating the removal of lactyl groups. Another significant mechanistic insight highlighted in our study is the role of SATB2 and HDAC3 in facilitating H3K9 delactylation. Co‐IP results demonstrated that HDAC3, a prominent “eraser” of histone lactylation, physically interacted with SATB2. Functionally, overexpression of HDAC3 recapitulated amplified the suppressive effects of SATB2 on LCN2 expression and H3K9 delactylation, while HDAC3 knockdown showed reversed effects. Overall, our results demonstrated that SATB2 recruits HDAC3 to catalyze the delactylation of histone H3K9 at the promoter of the oncogene LCN2, thereby repressing its transcription and impeding tumor growth and metastasis (Figure 8K).

The molecular pathway elucidated in this study, wherein SATB2 recruits HDAC3 to catalyze H3K9 delactylation and transcriptionally repress oncogene LCN2, provides several compelling avenues for clinical translation in NSCLC diagnosis and treatment. First, targeted gene therapy via SATB2‐stabilizing compounds for restoring its expression may be considered. Second, inhibition of LCN2 could be explored as adjunct therapies, particularly in SATB2‐low tumors. Moreover, the inverse correlation between SATB2 and LCN2 suggests their potential utility as a complementary prognostic biomarker for stratifying patients with higher risks for metastasis and poorer outcomes, potentiating more personalized surveillance options. In addition, given that lactate availability influences H3K9la levels, pharmacological treatments used to reduce lactate production might synergize with conventional chemotherapy or immunotherapy. Future clinical studies are warranted to evaluate whether tumors with low SATB2/high LCN2 expression benefit from interventions aimed at this pathway.

## 4 Experimental Methods

### 4.1 Cell Lines and Culture

The NSCLC cell lines used for this study (A549 (RRID:CVCL_0023), H1299 (RRID:CVCL_0060), PC‐9 (RRID:CVCL_B260), H1975 (RRID:CVCL_1511), H460 (RRID:CVCL_0459), and human embryonic kidney 293T (RRID:CVCL_0063) were purchased from ATCC. A549 cells were cultured in F12K (HyClone, USA) with 10% FBS (Gibco, USA) and 1% P/S (Solarbio, China). HEK293T cells were cultured in DMEM (HyClone, USA) with 10% fetal bovine serum (FBS) (Gibco, USA). H1975, H1299, H460 and PC‐9 cells were cultured in RPMI‐1640 medium (HyClone, USA) with 10% FBS (Gibco, USA). The culture conditions were 37°C and 5% CO^2^. All cells were authenticated to be mycoplasma‐free by STR profiling.

### 4.2 Cell Transfection

Transfection of cells with either siRNA or plasmid DNA was conducted using Lipofectamine 2000 reagent (Thermo Fisher Scientific, USA), in accordance with the manufacturer's protocol.

### 4.3 RNA Extraction and Quantitative Real‐Time PCR (qRT‐PCR)

Total RNA was isolated from cells or tissues with TRIzol reagent (Genestar, Shanghai, China) according to the manufacturer's instructions. The extracted RNA was reverse‐transcribed into cDNA using a commercial kit (Yeasen, Shanghai, China). Quantitative real‐time PCR (qRT‐PCR) was then performed employing a SYBR Green PCR kit (Yeasen, Shanghai, China). The primers are listed in Table S1. Gene expression levels were analyzed by the 2−^ΔΔCt^ method.

### 4.4 Protein Extraction and Western Blot

Protein extracts from cells or tissues, prepared using RIPA buffer (Pioneer, Xi'an, China) with protease inhibitors (HY‐K0010, MCE, China), were subjected to SDS‐PAGE (10‐12% gels) and transferred to PVDF membranes (Merck Millipore, Germany). Following a 1 h block in 5% skim milk, membranes were probed with primary antibodies overnight at 4 °C and then with HRP‐conjugated secondary antibodies for 1 h at room temperature. Target proteins were detected by enhanced chemiluminescence (ECL). Antibody details are provided in Table S2.

### 4.5 siRNA, Plasmid and Lentivirus

The siRNAs, overexpress plasmids and lentivirus were constructed by GenePharma (Shanghai, China). The siRNAs sequences were list in Table S1.

### 4.6 Transwell Migration and Invasion Assays

8.0‐µm 24‐Transwell chambers (Corning, USA) were used for transwell assays. The chambers used in the transwell invasion assay were precoated with Matrigel matrix (BD Biosciences, USA). Cells (4 × 10^4^) were seeded into the upper chambers., then the chambers were starved after 24 h. Following a 24 h incubation, the membranes were processed by fixing with formaldehyde and staining with 0.5% crystal violet (Beyotime, China) to visualize the cells that had migrated or invaded to the lower side, which were then counted using a Nikon inverted microscope.

### 4.7 Dual‐Luciferase Reporter Assay (Luciferase Reporter Assay)

The LCN2 promoter sequence (‐1700 to 100) or three copies of the MAR sequence (TATTCTTTATTATTTT) were inserted upstream of the promoter of pGL3 luciferase vector (Promega, Madison, WI, USA). The MAR‐containing reporter was co‐transfected with the SATB2 expression vector into HEK293 cells. The LCN2 promoter reporter construct was transfected into A549, H1299 and PC9 cells, which were then treated with 20 mm NaLa for 24 h. At 48 h after transfection, luciferase activity assay was measured using Dual Luciferase Reporter Gene Assay Kit (11402ES60, Yeasen, China) according to the manufacturer's instructions. Renilla luciferase activity was used as an internal standard.

### 4.8 Wound Healing Assay

Cells were seeded in six‐well plates and transfected. Following the creation of a scratch wound in the monolayer with a sterile pipette tip, the dislodged cells were removed by PBS washing. Wound areas were captured at 0, 24 and 48 h using a microscope.

### 4.9 Colony Forming Assay

A549, H1299, and PC9 cells were transfected and plated 24 h later in 12‐well plates (500 cells per well). Following a 10 d incubation, colonies were fixed with 4% paraformaldehyde, stained with crystal violet, and counted.

### 4.10 Analysis of Apoptosis

Cell apoptosis assay was performed by flow cytometry (FACSCalibur, BD, Biosciences, USA). According the manufacturer's instructions, cells by stained using Annexin V‐FITC/PI apoptosis detection kit (40302ES60, Yeasen, China).

### 4.11 RNA‐Seq

Total RNA was extracted from A549 cells stably expressing SATB2. The RNA extraction and RNA‐seq were carried out by OBiO Technology Corp., Ltd. (Shanghai, China). After extraction, the mRNA was captured and segmented. This was followed by reverse transcription, end‐repair, adaptor ligation, and PCR amplification. Finally, the sequencing library was purified using magnetic beads. Following quality control, sequencing was performed using an Illumina NovaSeq 6000 system, in accordance with Illumina protocols for 2×150 paired‐end sequencing.

### 4.12 CUT&Tag

The CUT&Tag assay was performed according to the manufacturer's instructions using the Hyperactive Universal CUT&Tag Assay Kit for Illumina Pro (TD904, Vazyme Biotech, Nanjing, China). Briefly, cells were bound to concanavalin A‐coated magnetic beads (ConA beads). The cell membrane was permeabilized with the nonionic detergent digitonin. The bead‐bound cells were then incubated sequentially with the H3K9la antibody, a secondary antibody, and the Hyperactive pA‐Tn5 Transposase. This procedure enables the Hyperactive pA‐Tn5 transposase to precisely cleave the DNA fragments bound by the target protein. Subsequently, the cleaved DNA fragments were ligated to Illumina P5 and P7 adapters by the Tn5 transposase, and the libraries were amplified via PCR using the corresponding primers. The PCR products were purified, and their quality was assessed using an Agilent 2100 Bioanalyzer (Agilent Technologies, USA). Finally, the libraries were sequenced on an Illumina NovaSeq 6000 platform to generate 150 bp paired‐end reads for subsequent analysis.

### 4.13 Glycolytic Process Evaluation

To evaluate the status of glycolysis, 2‐NBDG (ST1024, Beyotime, Nanjing, China) was used to determine glucose uptake ability, and the production of lactate was measured by Lactic Acid assay kit (A019‐2‐1, Nanjing Jiancheng Bioengineering Institute, China). All procedures were performed according to the manual.

### 4.14 Chromatin Immunoprecipitation (ChIP)

Cells were crosslinked in 1% formaldehyde for 15 min at 37°C and subsequently incubated with glycine (0.125 mol/L) for 30 min to stop crosslinking. Chromatin was fragmented via sonication, and the lysate was immunoprecipitated using Protein A/G Magnetic Beads (HY‐K0202, MCE, China) and antibodies against SATB2 at 4°C overnight. qRT‐PCR was performed after DNA elution and purification. The primer sequences used are listed in Table S1, and the antibodies used are listed in Table S2.

### 4.15 Protein Immunoprecipitation (IP)

Cells were lysed using RIPA buffer, then the lysate was incubated overnight with specific antibodies at 4°C. The next day, Protein A/G Magnetic Beads (HY‐K0202, MCE, China) were added to capture antibodies and proteins. The captured proteins then analyzed by immunoblot analysis.

### 4.16 Immunohistochemistry (IHC)

Paraffin‐embedded tissue sections were prepared from samples fixed in 4% formaldehyde. After deparaffinization, rehydration, antigen retrieval, and blocking, the sections were incubated sequentially with a primary antibody (4°C, overnight) and a secondary antibody (room temperature). Signal detection was performed using 3,3'‐diaminobenzidine (DAB) for visualization, with hematoxylin used as a counterstain. Images were acquired with a microscopic analysis system (Media Cybernetics, USA).

### 4.17 Hematoxylin‐Eosin Staining (HE Staining)

The tissue was fixed with 4% formaldehyde solution for 48 h, followed by paraffin embedding and sectioning. After dewaxing, the paraffin sections were stained with hematoxylin and eosin.

### 4.18 Immunofluorescence Assay

Cells were fixed with 4% paraformaldehyde and permeabilized with 0.1% Triton‐X. Later, 3% BSA solution was used for blocking. After incubation with primary antibodies and secondary antibodies, images were captured by fluorescence microscope (Leica STELLARIS 5, Germany). The nuclei were stained with DAPI.

### 4.19 Human Ethics

The use of patient tissue samples in this study was approved by the Ethics Committee of the First Affiliated Hospital of Xi'an Jiaotong University (LLSBPJ‐2025‐228). Informed consent was obtained from all participants.

### 4.20 In Vivo Mouse Model

#### 4.20.1 Subcutaneous Tumorigenesis Model

A549 cells (1 × 10^6^) were suspended in PBS and injected subcutaneously into 5‐week‐old male nude mice. The parameters of subcutaneous tumors were recorded every 7 d. Of note, the volume of the tumor was speculated by the formula below: tumor volume  =  0.5 × length × width^2^ (mm^3^). After 21 d, tumors were harvested from sacrificed mice and weighed.

#### 4.20.2 Tail Vein‐lung Metastasis Model

A549 cells (1 × 10^6^) were suspended in PBS and injected via the tail vein into male ‐week‐old nude BALB/c mice. Six weeks later, the mice were sacrificed, and the lung tissues were dissected. The lung tissues were collected for HE staining. Mice were bred in the Animal Research Center of Xi'an Jiaotong University. The Guidelines for the Care and Use of Laboratory Animals of the National Institute of Health in China were followed in terms of both animal care and experimentation. All animal experiments were approved by the Ethics Committee of Xi'an Jiaotong University (Approval Number: XJTUAE2025‐386).

### 4.21 Statistical Analysis

Statistical analyses were performed using GraphPad Prism 9.0.0. All experiments were repeated at least in triplicate unless otherwise stated. Student's *t*‐test (two‐tailed) (for equal variance) and Welch's *t*‐test (for unequal variance) were used for comparisons between two independent groups. One‐way ANOVA followed by multiple comparisons using Dunnett's test was performed to analyze differences among more than two groups. The data in this study are presented as the mean ± standard deviation (SD). *P* value < 0.05 was regarded as statistically significant.

## Author Contributions

Q.Y.C. and S.D.Q. designed the study and conceptually supervised the project. T.W. performed all experiments. L.H.Y., S.C., Y.X.L., J.N.F., P.W., B.B.G., and X.G.X. assisted with in vitro and in vivo experiments, as well as interpretation of data. H.S. contributed to the design of the experiments and critical revision of the manuscript. T.W. wrote the manuscript. Q.Y.C., S.D.Q., and H.S. revised the manuscript. All authors approved the final version of the article.

## Funding

This study was supported by the National Natural Science Foundation of China 82302973, 82373182, and Xi'an Jiaotong University “Young Talent Support Plan” No. YX6J010.

## Conflicts of Interest

The authors declare no conflicts of interest.

## Supporting information




**Supporting File 1**: advs74017‐sup‐0001‐SuppMat.docx.

**Supporting File 2**: advs74017‐sup‐0002‐SuppTables.xlsx.

## Data Availability

The data that support the findings of this study are available on request from the corresponding author. The data are not publicly available due to privacy or ethical restrictions.

## References

[1] J. Bi , J. Tuo , Y. Xiao , et al., “Observed and Relative Survival Trends of Lung Cancer: a Systematic Review of Population‐Based Cancer Registration Data,” Thoracic Cancer 15, no. 2 (2024): 142–151, 10.1111/1759-7714.15170. 37986711 PMC10788469

[2] H. Sung , J. Ferlay , R. L. Siegel , et al., “Global Cancer Statistics 2020: GLOBOCAN Estimates of Incidence and Mortality Worldwide for 36 Cancers in 185 Countries,” CA: A Cancer Journal for Clinicians 71, no. 3 (2021): 209–249, 10.3322/caac.21660. 33538338

[3] G. Cheng , C. Tian , W. Wang , Y. Zhou , X. Wang , and L. Zhang , “Advances in Research on SATB2 and Its Role in Tumor Development,” Cell & Bioscience 15, no. 1 (2025): 111, 10.1186/s13578-025-01439-1. 40722111 PMC12302682

[4] W. Dong , Y. Chen , N. Qian , et al., “SATB2 knockdown Decreases Hypoxia‑Induced Autophagy and Stemness in Oral Squamous Cell Carcinoma,” Oncology Letters 20, no. 1 (2020): 794–802, 10.3892/ol.2020.11589. 32566006 PMC7285822

[5] E. A. Turovsky , M. V. Turovskaya , E. I. Fedotova , A. A. Babaev , V. S. Tarabykin , and E. G. Varlamova , “Role of Satb1 and Satb2 Transcription Factors in the Glutamate Receptors Expression and Ca^2+^ Signaling in the Cortical Neurons in Vitro,” International Journal of Molecular Sciences 22, no. 11 (2021): 5968, 10.3390/ijms22115968. 34073140 PMC8198236

[6] Q. Y. Chen , T. Des Marais , and M. Costa , “Deregulation of SATB2 in Carcinogenesis with Emphasis on miRNA‐Mediated Control,” Carcinogenesis 40, no. 3 (2019): 393–402, 10.1093/carcin/bgz020. 30916759 PMC6514447

[7] Y. Ma , H. Zhang , L. Fei , et al., “SATB2 suppresses Non‐Small Cell Lung Cancer Invasiveness by G9a,” Clinical and Experimental Medicine 18, no. 1 (2018): 37–44, 10.1007/s10238-017-0464-3. 28667416

[8] H. Kucuksayan , O. N. Ozes , and H. Akca , “Downregulation of SATB2 Is Critical for Induction of Epithelial‐to‐Mesenchymal Transition and Invasion of NSCLC Cells,” Lung Cancer 98 (2016): 122–129, 10.1016/j.lungcan.2016.05.032. 27393518

[9] H. Kucuksayan and H. Akca , “The Crosstalk between p38 and Akt Signaling Pathways Orchestrates EMT by Regulating SATB2 Expression in NSCLC Cells,” Tumor Biology 39, no. 9 (2017): 101042831770621, 10.1177/1010428317706212. 28937318

[10] F. Yu , M. Liang , Y. Huang , W. Wu , B. Zheng , and C. Chen , “Hypoxic Tumor‐Derived Exosomal miR‐31‐5p Promotes Lung Adenocarcinoma Metastasis by Negatively Regulating SATB2‐Reversed EMT and Activating MEK/ERK Signaling,” Journal of Experimental & Clinical Cancer Research 40, no. 1 (2021): 179, 10.1186/s13046-021-01979-7. 34074322 PMC8167983

[11] Z. Su , M. Chen , R. Ding , L. Shui , Q. Zhao , and W. Luo , “Long Non‑Coding RNA HCG11 Suppresses the Malignant Phenotype of Non‑Small Cell Lung Cancer Cells by Targeting a miR‑875/SATB2 Axis,” Molecular Medicine Reports 24, no. 2 (2021): 552, 10.3892/mmr.2021.12191. 34080031 PMC8188752

[12] J. Wang , Y. Lu , H. Ding , et al., “The miR‐875‐5p Inhibits SATB2 to Promote the Invasion of Lung Cancer Cells,” Gene 644 (2018): 13–19, 10.1016/j.gene.2017.11.066. 29196257

[13] Z. Wang , L. Zhou , B. Chen , et al., “microRNA‐660 Enhances Cisplatin Sensitivity via Decreasing SATB2 Expression in Lung Adenocarcinoma,” Genes 14, no. 4 (2023): 911, 10.3390/genes14040911. 37107669 PMC10137726

[14] J. Gu , G. Wang , H. Liu , and C. Xiong , “SATB 2 Targeted by Methylated miR‐34c‐5p Suppresses Proliferation and Metastasis Attenuating the Epithelial‐Mesenchymal Transition in Colorectal Cancer,” Cell Proliferation 51, no. 4 (2018): 12455, 10.1111/cpr.12455. PMC6528935 29701273

[15] X. Lv , Y. Lv , and X. Dai , “Lactate, Histone Lactylation and Cancer Hallmarks,” Expert Reviews in Molecular Medicine 25 (2023): 7, 10.1017/erm.2022.42. 36621008

[16] D. Zhang , Z. Tang , H. Huang , et al., “Metabolic Regulation of Gene Expression by Histone Lactylation,” Nature 574, no. 7779 (2019): 575–580, 10.1038/s41586-019-1678-1. 31645732 PMC6818755

[17] J. Chen , Z. Huang , Y. Chen , et al., “Lactate and Lactylation in Cancer,” Signal Transduction and Targeted Therapy 10, no. 1 (2025): 38, 10.1038/s41392-024-02082-x. 39934144 PMC11814237

[18] C. T. Hensley , B. Faubert , Q. Yuan , et al., “Metabolic Heterogeneity in Human Lung Tumors,” Cell 164, no. 4 (2016): 681–694, 10.1016/j.cell.2015.12.034. 26853473 PMC4752889

[19] Y. Ji , Z. Xu , L. Tang , et al., “O‐GlcNAcylation of YBX1 Drives a Glycolysis‐Histone Lactylation Feedback Loop in Hepatocellular Carcinoma,” Cancer Letters 631 (2025): 217957, 10.1016/j.canlet.2025.217957. 40721081

[20] J. Jiang , D. Huang , Y. Jiang , et al., “Lactate Modulates Cellular Metabolism through Histone Lactylation‐Mediated Gene Expression in Non‐Small Cell Lung Cancer,” Frontiers in Oncology 11 (2021): 647559, 10.3389/fonc.2021.647559. 34150616 PMC8208031

[21] T. Peng , F. Sun , J. Yang , et al., “Novel Lactylation‐Related Signature to Predict Prognosis for Pancreatic Adenocarcinoma,” World Journal of Gastroenterology 30, no. 19 (2024): 2575–2602, 10.3748/wjg.v30.i19.2575. 38817665 PMC11135411

[22] L. Sun , Y. Zhang , B. Yang , et al., “Lactylation of METTL16 Promotes Cuproptosis via m6A‐Modification on FDX1 mRNA in Gastric Cancer,” Nature Communications 14, no. 1 (2023): 6523, 10.1038/s41467-023-42025-8. PMC10589265 37863889

[23] J. Yu , P. Chai , M. Xie , et al., “Histone Lactylation Drives Oncogenesis by Facilitating m6A Reader Protein YTHDF2 Expression in Ocular Melanoma,” Genome Biology 22, no. 1 (2021): 85, 10.1186/s13059-021-02308-z. 33726814 PMC7962360

[24] M. Zhao , Y. Qian , L. He , et al., “Lactate‐Mediated Histone Lactylation Promotes Melanoma Angiogenesis via IL‐33/ST2 Axis,” Cell Death & Disease 16, no. 1 (2025): 701, 10.1038/s41419-025-08023-y. 41053006 PMC12501017

[25] C. Zhang , L. Zhou , M. Zhang , et al., “H3K18 Lactylation Potentiates Immune Escape of Non–Small Cell Lung Cancer,” Cancer Research 84, no. 21 (2024): 3589–3601, 10.1158/0008-5472.can-23-3513. 39137401

[26] L. Shi , B. Li , J. Tan , et al., “Exosomal lncRNA Mir100hg from Lung Cancer Stem Cells Activates H3K14 Lactylation to Enhance Metastatic Activity in Non‐Stem Lung Cancer Cells,” Journal of Nanobiotechnology 23, no. 1 (2025): 156, 10.1186/s12951-025-03198-0. 40022086 PMC11869636

[27] M. Liu , L. Gu , Y. Zhang , et al., “LKB1 Inhibits Telomerase Activity Resulting in Cellular Senescence through Histone Lactylation in Lung Adenocarcinoma,” Cancer Letters 595 (2024): 217025, 10.1016/j.canlet.2024.217025. 38844063

[28] W. Duan , W. Liu , S. Xia , et al., “Warburg Effect Enhanced by AKR1B10 Promotes Acquired Resistance to Pemetrexed in Lung Cancer‐Derived Brain Metastasis,” Journal of Translational Medicine 21, no. 1 (2023): 547, 10.1186/s12967-023-04403-0. 37587486 PMC10428599

[29] S. Zhao , J. Cheng , G. Zhou , et al., “Histone Lactylation‐Driven YTHDF2 Promotes Non‐Small Cell Lung Cancer Cell Glycolysis and Stemness by Recognizing m6A Modification of SFRP2,” Biochemical Pharmacology 240 (2025): 117097, 10.1016/j.bcp.2025.117097. 40619016

[30] C. Moreno‐Yruela , D. Zhang , W. Wei , et al., “Class I Histone Deacetylases (HDAC1–3) Are Histone Lysine Delactylases,” Science Advances 8, no. 3 (2022): abi6696, 10.1126/sciadv.abi6696. PMC8769552 35044827

[31] Z. Zhang , S. Ren , W. Yang , et al., “AARS2‐Catalyzed Lactylation Induces Follicle Development and Premature Ovarian Insufficiency,” Cell Death Discovery 11, no. 1 (2025): 209, 10.1038/s41420-025-02501-0. 40301335 PMC12041370

[32] C. Baranek , M. Dittrich , S. Parthasarathy , et al., “Protooncogene Ski Cooperates with the Chromatin‐Remodeling Factor Satb2 in Specifying Callosal Neurons,” Proceedings of the National Academy of Sciences 109, no. 9 (2012): 3546–3551, 10.1073/pnas.1108718109. PMC3295291 22334647

[33] O. Britanova , C. de Juan Romero , A. Cheung , et al., “Satb2 is a Postmitotic Determinant for Upper‐Layer Neuron Specification in the Neocortex,” Neuron 57, no. 3 (2008): 378–392, 10.1016/j.neuron.2007.12.028. 18255031

[34] W. Gu , X. Huang , P. N. P. Singh , et al., “A MTA2‐SATB2 Chromatin Complex Restrains Colonic Plasticity toward Small Intestine by Retaining HNF4A at Colonic Chromatin,” Nature Communications 15, no. 1 (2024): 3595, 10.1038/s41467-024-47738-y. PMC11055869 38678016

[35] A. B. Gyorgy , M. Szemes , C. de Juan Romero , V. Tarabykin , and D. V. Agoston , “SATB2 Interacts with Chromatin‐Remodeling Molecules in Differentiating Cortical Neurons,” European Journal of Neuroscience 27, no. 4 (2008): 865–873, 10.1111/j.1460-9568.2008.06061.x. 18333962

[36] F. Li , C. Yan , Y. Yao , et al., “Transcription Factor SATB2 Regulates Skeletal Muscle Cell Proliferation and Migration via HDAC4 in Pigs,” Genes (Basel) 15, no. 1 (2024): 15, 10.3390/genes15010065. PMC10815226 38254955

[37] X. Huang , Q. Chen , W. Luo , et al., “SATB2: A Versatile Transcriptional Regulator of Craniofacial and Skeleton Development, Neurogenesis and Tumorigenesis, and Its Applications in Regenerative Medicine,” Genes & Diseases 9, no. 1 (2022): 95–107, 10.1016/j.gendis.2020.10.003. 35005110 PMC8720659

[38] R. Naik and S. Galande , “SATB family Chromatin Organizers as Master Regulators of Tumor Progression,” Oncogene 38, no. 12 (2019): 1989–2004, 10.1038/s41388-018-0541-4. 30413763

[39] J. Yang , Y. Li , Y. Tang , L. Yang , C. Guo , and C. Peng , “Spatial Transcriptome Reveals the Region‐Specific Genes and Pathways Regulated by Satb2 in Neocortical Development,” BMC Genomics 25, no. 1 (2024): 757, 10.1186/s12864-024-10672-w. 39095712 PMC11297773

[40] J. Cai , P. Zhang , Y. Cai , et al., “Lactylation‐Driven NUPR1 Promotes Immunosuppression of Tumor‐Infiltrating Macrophages in Hepatocellular Carcinoma,” Advanced Science 12, no. 20 (2025): 2413095, 10.1002/advs.202413095. 40305758 PMC12120759

[41] M. Gao , M. Wang , S. Zhou , et al., “Machine Learning‐Based Prognostic Model of Lactylation‐Related Genes for Predicting Prognosis and Immune Infiltration in Patients with Lung Adenocarcinoma,” Cancer Cell International 24, no. 1 (2024): 400, 10.1186/s12935-024-03592-y. 39696439 PMC11656871

[42] H. Wang , M. Xu , T. Zhang , et al., “PYCR1 promotes Liver Cancer Cell Growth and Metastasis by Regulating IRS1 Expression through Lactylation Modification,” Clinical and Translational Medicine 14, no. 10 (2024): 70045, 10.1002/ctm2.70045. PMC11488319 39422696

[43] S. Wei , J. Zhang , R. Zhao , et al., “Histone Lactylation Promotes Malignant Progression by Facilitating USP39 Expression to Target PI3K/AKT/HIF‐1α Signal Pathway in Endometrial Carcinoma,” Cell Death Discovery 10, no. 1 (2024): 121, 10.1038/s41420-024-01898-4. 38459014 PMC10923933

[44] W. Tao , A. Zhang , K. Zhai , et al., “SATB2 Drives Glioblastoma Growth by Recruiting CBP to Promote FOXM1 Expression in Glioma Stem Cells,” EMBO Molecular Medicine 12, no. 12 (2020): 12291, 10.15252/emmm.202012291. PMC7721366 33124191

[45] D. Ding , W. Shang , K. Shi , et al., “FTO/m6A Mediates miR‐138‐5p Maturation and Regulates Gefitinib Resistance of Lung Adenocarcinoma Cells by miR‐138‐5p/LCN2 Axis,” BMC Cancer 24, no. 1 (2024): 1270, 10.1186/s12885-024-13036-5. 39394098 PMC11470737

[46] A. Li , K. Zhang , J. Zhou , et al., “Bioinformatics and Experimental Approach Identify Lipocalin 2 as a Diagnostic and Prognostic Indicator for Lung Adenocarcinoma,” International Journal of Biological Macromolecules 272, no. Pt 2 (2024): 132797, 10.1016/j.ijbiomac.2024.132797. 38848833

[47] D. Wang , X. Li , D. Jiao , et al., “LCN2 secreted by Tissue‐Infiltrating Neutrophils Induces the Ferroptosis and Wasting of Adipose and Muscle Tissues in Lung Cancer Cachexia,” Journal of Hematology & Oncology 16, no. 1 (2023): 30, 10.1186/s13045-023-01429-1. 36973755 PMC10044814

[48] J. Zhang , Q. Xu , and G. Sun , “Lipocalin‐2 Promotes NSCLC Progression by Activating the JAK2/STAT3 Signaling Pathway,” Journal of Translational Medicine 23, no. 1 (2025): 419, 10.1186/s12967-025-06418-1. 40211270 PMC11987316

[49] Q. Zhou , W. Zhang , J. Huang , and W. Hu , “ZEB1‐Upregulated LCN2 via Transcription Regulation Affects Ferroptosis and Malignant Progression in Non‐Small Cell Lung Cancer,” Pathology—Research and Practice 274 (2025): 156154, 10.1016/j.prp.2025.156154. 40782465

[50] M. B. Gonzatti , J. C. Hintzen , I. Sharma , et al., “Class I Histone Deacetylases Catalyze Lysine Lactylation,” Journal of Biological Chemistry 301, no. 10 (2025): 110602, 10.1016/j.jbc.2025.110602. 40835008 PMC12624779

[51] X. He , Y. Li , J. Li , et al., “HDAC2‐Mediated METTL3 Delactylation Promotes DNA Damage Repair and Chemotherapy Resistance in Triple‐Negative Breast Cancer,” Advanced Science 12, no. 14 (2025): 2413121, 10.1002/advs.202413121. 39950833 PMC11984901

[52] X. Li , M. Chen , X. Chen , et al., “TRAP1 drives Smooth Muscle Cell Senescence and Promotes Atherosclerosis via HDAC3‐Primed Histone H4 Lysine 12 Lactylation,” European Heart Journal 45, no. 39 (2024): 4219–4235, 10.1093/eurheartj/ehae379. 39088352 PMC11481199

[53] J. Li , L. Feng , L. Zhang , et al., “Saikosaponin D Mitigates Radioresistance in Triple‐Negative Breast Cancer by Inducing MRE11 De‐Lactylation via HIF1α/HDAC5 Pathway,” Theranostics 15, no. 17 (2025): 8935–8951, 10.7150/thno.113517. 40963916 PMC12439262

[54] H. Rho , A. R. Terry , C. Chronis , and N. Hay , “Hexokinase 2‐Mediated Gene Expression via Histone Lactylation Is Required for Hepatic Stellate Cell Activation and Liver Fibrosis,” Cell Metabolism 35, no. 8 (2023): 1406–1423.e8, 10.1016/j.cmet.2023.06.013. 37463576 PMC11748916

[55] S. Sun , Z. Xu , L. He , et al., “Metabolic Regulation of Cytoskeleton Functions by HDAC6‐Catalyzed α‐Tubulin Lactylation,” Nature Communications 15, no. 1 (2024): 8377, 10.1038/s41467-024-52729-0. PMC11437170 39333081

[56] Y. Zou , M. Cao , M. Tai , et al., “A Feedback Loop Driven by H4K12 Lactylation and HDAC3 in Macrophages Regulates Lactate‐Induced Collagen Synthesis in Fibroblasts via the TGF‐β Signaling,” Advanced Science 12, no. 13 (2025): 2411408, 10.1002/advs.202411408. 39945346 PMC11967864

[57] H. Xu , L. Li , S. Wang , et al., “Royal Jelly Acid Suppresses Hepatocellular Carcinoma Tumorigenicity by Inhibiting H3 Histone Lactylation at H3K9la and H3K14la Sites,” Phytomedicine 118 (2023): 154940, 10.1016/j.phymed.2023.154940. 37453194

[58] Q. Yue , Z. Wang , Y. Shen , et al., “Histone H3K9 Lactylation Confers Temozolomide Resistance in Glioblastoma via LUC7L2‐Mediated MLH1 Intron Retention,” Advanced Science 11, no. 19 (2024): 2309290, 10.1002/advs.202309290. 38477507 PMC11109612

[59] Y. Zang , A. Wang , J. Zhang , et al., “Hypoxia Promotes Histone H3K9 Lactylation to Enhance LAMC2 Transcription in Esophageal Squamous Cell Carcinoma,” iScience 27, no. 7 (2024): 110188, 10.1016/j.isci.2024.110188. 38989468 PMC11233973

[60] C. Zhang , R. Yu , S. Li , et al., “KRAS Mutation Increases Histone H3 Lysine 9 Lactylation (H3K9la) to Promote Colorectal Cancer Progression by Facilitating Cholesterol Transporter GRAMD1A Expression,” Cell Death and Differentiation 32 (2025): 2225—2238, 10.1038/s41418-025-01533-4. 40707783 PMC12669710

[61] H. Yan , Q. He , Y. Gao , et al., “SLC4A7 suppresses Lung Adenocarcinoma Oncogenesis by Reducing Lactate Transport and Protein Lactylation,” International Journal of Oncology 66, no. 5 (2025): 1–12, 10.3892/ijo.2025.5739. 40084702 PMC12002671

[62] K. J. Meade , F. Sanchez , A. Aguayo , et al., “Secretomes from Metastatic Breast Cancer Cells, Enriched for a Prognostically Unfavorable LCN2 Axis, Induce Anti‐Inflammatory MSC Actions and a Tumor‐Supportive Premetastatic Lung,” Oncotarget 10, no. 32 (2019): 3027–3039, 10.18632/oncotarget.26903. 31105883 PMC6508963

[63] R. K. Mongre , S. S. Sodhi , N. Sharma , et al., “Epigenetic Induction of Epithelial to Mesenchymal Transition by LCN2 Mediates Metastasis and Tumorigenesis, Which Is Abrogated by NF‐κB Inhibitor BRM270 in a Xenograft Model of Lung Adenocarcinoma,” International Journal of Oncology 48, no. 1 (2016): 84–98, 10.3892/ijo.2015.3245. 26573874 PMC4734607

[64] C. Shi , C. Wang , Z. Fu , et al., “Lipocalin 2 (LCN2) Confers Acquired Resistance to Almonertinib in NSCLC through LCN2‐MMP‐9 Signaling Pathway,” Pharmacological Research 201 (2024): 107088, 10.1016/j.phrs.2024.107088. 38295916

[65] M. Shiiba , K. Saito , K. Fushimi , et al., “Lipocalin‐2 Is Associated with Radioresistance in Oral Cancer and Lung Cancer Cells,” International Journal of Oncology 42, no. 4 (2013): 1197–1204, 10.3892/ijo.2013.1815. 23403985

[66] X. Song , S. Xu , D. Song , et al., “TGFB1/CXCL5 axis Regulation by LCN2 Overexpression: A Promising Strategy to Inhibit Colorectal Cancer Metastasis and Enhance Prognosis,” Frontiers in Immunology 16 (2025): 1548635, 10.3389/fimmu.2025.1548635. 40313933 PMC12043584

