# Supplementary material for: SATB2 Mediates H3K9 Delactylation by Recruiting HDAC3 to Repress LCN2 and Inhibit Lung Tumor Growth and Metastasis
Source: Adv Sci (Weinh). 2026 Feb 25;13(19):e22996. doi: 10.1002/advs.202522996 (PMC13045397; doi:10.1002/advs.202522996)
Supplement: Supplementary file 1 — Supporting File 1: advs74017‐sup‐0001‐SuppMat.docx. [file ADVS-13-e22996-s001.docx]

**Supplementary figures**

**
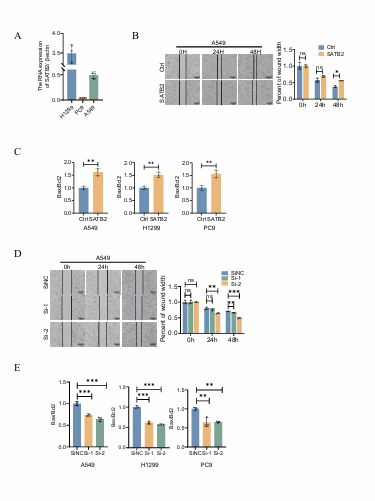
**

**Figure S1. A.** mRNA expression levels of SATB2 in LUAD cells. **B.** The migration ability of A549 cells overexpressing SATB2 was evaluated at 0, 24, and 48 hours using a wound healing assay. **C.** The Bax/Bcl-2 protein expression ratio in LUAD cells following SATB2 overexpression. **D.** The migration ability of A549 cells with knockdown of SATB2 was evaluated at 0, 24, and 48 hours using a wound healing assay. **E.** The Bax/Bcl-2 protein expression ratio in LUAD cells following SATB2 knockdown.


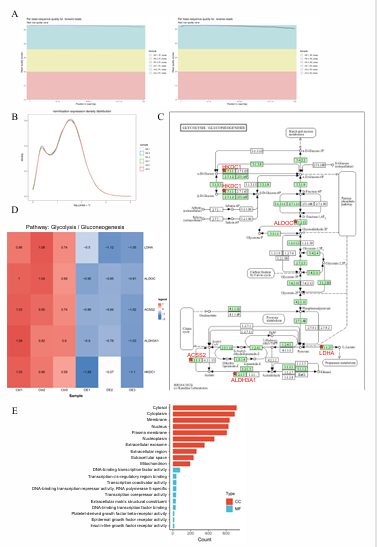


**Figure S2. A.** The base quality score of the reads. Left is base quality score for Reads 1, and right is for Reads 2. **B.** Sample expression distribution. **C.** The pathway related to glucose metabolism. The differential expression genes in the glycolysis/gluconeogenesis pathway based on RNA-seq data from A549 cells stably expressing SATB2. **D.** The pathway enrichment of up-regulated (left) and down-regulated (right) genes based on RNA-seq data. **E.** GO enrichment analysis of molecular function and cellular component based on RNA-seq data. Heatmap and bar blot were plotted using Hiplot Pro (<https://hiplot.com.cn/>), a comprehensive web service for biomedical data analysis and visualization.


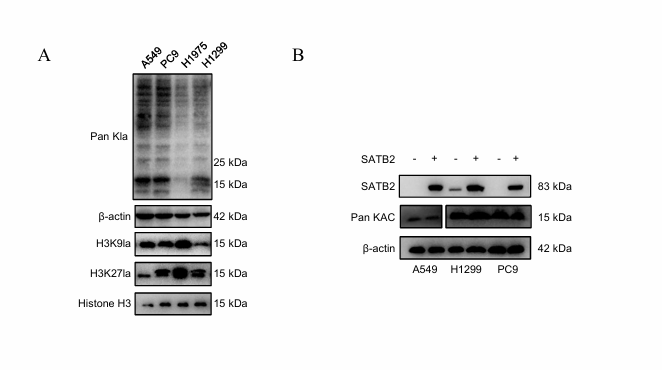


**Figure S3. A.** The protein level of Pan Kla, H3K9la and H3K27la in cells were measured by western blotting assays. **B.** The protein level of Pan Kac was measured by western blotting analyses.


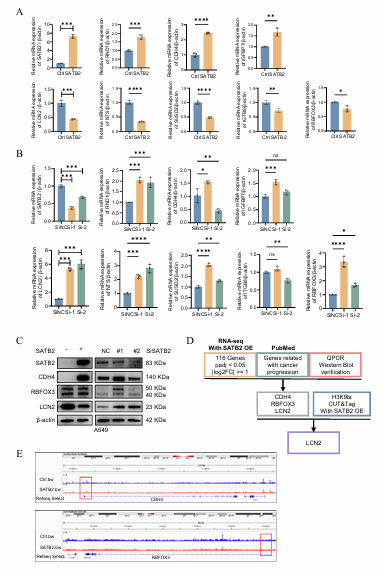


**Figure S4.** **A-B.** The results of RNA-seq were validated by qRT-PCR in in A549 cells with SATB2 overexpression **(A)** and knockdown **(B)**. **C.** The results of RNA-seq were validated by western blotting assays in in A549 cells with SATB2 overexpression and knockdown. **D.** Flow diagram showing the downstream target of H3K9la. **E.** IGV views of H3K9la CUT&Tag sequence data on LCN2.


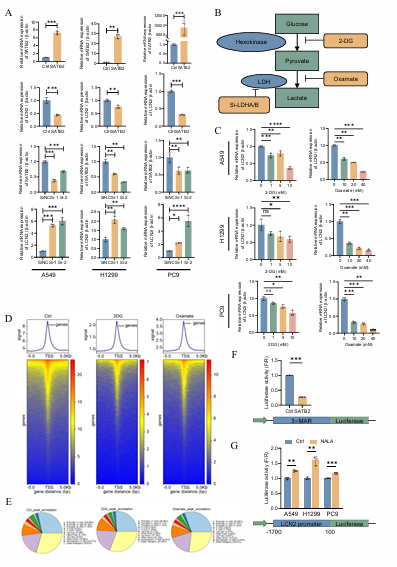


**Figure S5. A.** qRT-PCR analysis of LCN2 expression in SATB2-overexpression and SATB2-silenced cells. **B.** A schematic illustration of glycolysis is presented, and the methods used to inhibit the production of lactate and histone lactylation in this research are indicated. **C.** qRT-PCR analysis of LCN2 expression in cells with glycolysis inhibitors. **D-E.** CUT&Tag was performed with H3K9la antibodies in A549 cells with glycolysis inhibitors treated. The heatmap showed the distribution of H3K9la peaks in the transcriptional start site (TSS) **(D)**. The distribution of H3K9la on the genome **(E)**. **F.** Luciferase activity in HEK-293 cells was measured 48 h after transfection. **G.** Luciferase activity in LUAD cells was measured 48 h after transfection.


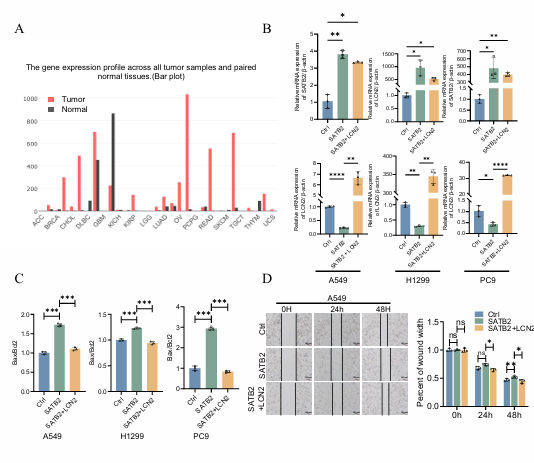


**Figure S6.** **A.** The expression of LCN2 mRNA in different cancer types from TCGA database. **B.** qRT-PCR analysis of SATB2 and LCN2 gene expression under LCN2 rescue. **C.** The migration ability of A549 cells with LCN2 rescue was evaluated at 0, 24, and 48 hours using a wound healing assay. **D.** The Bax/Bcl-2 protein expression ratio in LUAD cells under LCN2 rescue.

**
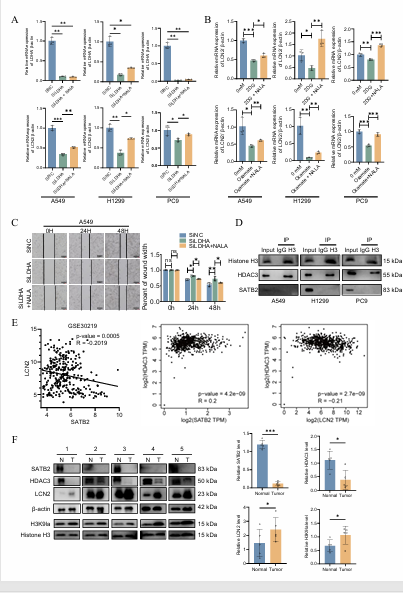
**

**Figure S7.** **A.** qRT-PCR analysis of LCN2 gene expression under silence LDHA and supplement with sodium lactate. **B.** qRT-PCR analysis of LCN2 gene expression under glycolysis inhibitors and sodium lactate supplementation. **C.** The migration ability of A549 cells under silence LDHA and supplement with sodium lactate was evaluated at 0, 24, and 48 hours using a wound healing assay. **D.** Endogenous immunoprecipitation (IP) with anti-H3 in the A549, H1299 and PC9 cells followed by western blotting. IgG was used as a negative control. **E.** Correlation analysis of SATB2 and LCN2 expression (GSE30219. n=293, Pearson r). Correlation analysis of HDAC3 expression with STAB2 or with LCN2 based on the RNA-seq results from TCGA database (Spearman r). **F.** Western blotting analysis of SATB2, HDAC3, LCN2 and H3K9la levels in 5 matched LUAD tissues (T) and adjacent normal tissues (N) (Paired *t*-test).
